# Production and Role of Hormones During Interaction of *Fusarium* Species With Maize (*Zea mays* L.) Seedlings

**DOI:** 10.3389/fpls.2018.01936

**Published:** 2019-01-11

**Authors:** Josef Vrabka, Eva-Maria Niehaus, Martin Münsterkötter, Robert H. Proctor, Daren W. Brown, Ondřej Novák, Aleš Pěnčik, Danuše Tarkowská, Kristýna Hromadová, Michaela Hradilová, Jana Oklešt’ková, Liat Oren-Young, Yifat Idan, Amir Sharon, Marcel Maymon, Meirav Elazar, Stanley Freeman, Ulrich Güldener, Bettina Tudzynski, Petr Galuszka, Veronique Bergougnoux

**Affiliations:** ^1^Department of Molecular Biology, Centre of the Region Haná for Biotechnological and Agricultural Research, Faculty of Science, Palacký University, Olomouc, Czechia; ^2^Institut für Biologie und Biotechnologie der Pflanzen, Molecular Biology and Biotechnology of Fungi, Westfälische Wilhelms-Universität Münster, Münster, Germany; ^3^Functional Genomics and Bioinformatics, Sopron University, Sopron, Hungary; ^4^National Center for Agricultural Utilization Research, United States Department of Agriculture, Peoria, IL, United States; ^5^Institute of Experimental Botany, Czech Academy of Sciences, Olomouc, Czechia; ^6^Department of Metabolomics, Centre of the Region Haná for Biotechnological and Agricultural Research, Faculty of Science, Palacký University, Olomouc, Czechia; ^7^Department of Molecular Biology and Ecology of Plants, Tel Aviv University, Tel Aviv, Israel; ^8^Department of Plant Pathology and Weed Research, Agricultural Research Organization (ARO), The Volcani Center, Rishon LeZion, Israel; ^9^Department of Bioinformatics, TUM School of Life Sciences Weihenstephan, Technical University of Munich, Munich, Germany

**Keywords:** auxin, cytokinin, *Fusarium*, host–pathogen interaction, gibberellin, mango malformation disease (MMD)

## Abstract

It has long been known that hormones affect the interaction of a phytopathogen with its host plant. The pathogen can cause changes in plant hormone homeostasis directly by affecting biosynthesis or metabolism in the plant or by synthesizing and secreting the hormone itself. We previously demonstrated that pathogenic fungi of the *Fusarium* species complex are able to produce three major types of hormones: auxins, cytokinins, and gibberellins. In this work, we explore changes in the levels of these hormones in maize and mango plant tissues infected with *Fusarium*. The ability to produce individual phytohormones varies significantly across *Fusarium* species and such differences likely impact host specificity inducing the unique responses noted *in planta* during infection. For example, the production of gibberellins by *F. fujikuroi* leads to elongated rice stalks and the suppression of gibberellin biosynthesis in plant tissue. Although all *Fusarium* species are able to synthesize auxin, sometimes by multiple pathways, the ratio of its free form and conjugates in infected tissue is affected more than the total amount produced. The recently characterized unique pathway for cytokinin *de novo* synthesis in *Fusarium* appears silenced or non-functional in all studied species during plant infection. Despite this, a large increase in cytokinin levels was detected in *F. mangiferae* infected plants, caused likely by the up-regulation of plant genes responsible for their biosynthesis. Thus, the accumulation of active cytokinins may contribute to mango malformation of the reproductive organs upon infection of mango trees. Together, our findings provide insight into the complex role fungal and plant derived hormones play in the fungal–plant interactions.

## Introduction

The genus *Fusarium* is a filamentous fungus found readily in soil around the world and associated with multiple crop species. Although it can interact with plants as an endophyte, its growth as a biotroph, hemibiotroph, or necrotroph cause significant agronomic losses worldwide. Currently, the genus *Fusarium* is divided into 20 species complexes and nine monotypic lineages (O’Donnell et al., 2013). Species from the *Fusarium fujikuroi* complex (FFC) are best known for their ability to induce diseases such as “bakanae” in rice (Matic et al., 2017), ear and stalk rot in maize (Presello et al., 2008), pitch canker in pine (Gordon, 2006) and mango malformation disease (MMD) in mango (Freeman et al., 2014). Some disease symptoms can be clearly linked to hormone production by the fungi. In fact, culture filtrate of *Fusarium fujikuroi* (*Gibberella fujikuroi*) was the first source from which gibberellins (GAs) were isolated and identified several decades ago (Hedden and Sponsel, 2015). It was only later discovered that GAs are ubiquitous plant hormones that promote normal stem elongation. The contribution of additional GAs to infected rice plants by *F. fujikuroi* leads to abnormally long stems which is the typical “bakanae” symptom observed. The gene cluster responsible for GA synthesis in *F. fujikuroi* has been extensively characterized (Tudzynski and Hölter, 1998).

The production of other hormones such as cytokinins (CKs), auxins and ethylene by fusaria was first suggested over 35 years ago based on their detection in culture filtrates (Mańka, 1980; Van Staden and Nicholson, 1989; Thakur and Vyas, 1983). CKs can be produced by the tRNA decay pathway, which is conserved in almost all living organisms, or by *de novo* synthesis. The tRNA pathway was shown to contribute to CK content in some fungi including *Claviceps purpurea* (Chanclud et al., 2016; Hinsch et al., 2016; Morrison et al., 2017). Recently, we found evidence for possible *de novo* CK synthesis by members of the FFC based on whole genome sequence analysis (Niehaus et al., 2016). We identified two homologous gene clusters, designated CK1 and CK2 located near the GA gene cluster. Each cluster contains two genes: *IPTLOG* and *P450*. *IPTLOG* codes for an enzyme with dual activity: the isopentenyl transferase domain (IPT) is responsible for the conjugation of dimethylallyl pyrophosphate with ATP to form a CK precursor, which is then hydrolyzed by the phosphoribohydrolase domain (*LOG*) to form the CK isopentenyladenine (iP). *P450* codes for a putative cytochrome P450 monooxygenase which catalyzes the hydroxylation of iP to form the CK *trans-*zeatin (*t*Z; Hinsch et al., 2015).

The major auxin indole-3-acetic acid (IAA) can be produced by at least three pathways. The indole-3-acetamide (IAM) pathway, present in all FFC species, converts tryptophan into IAA via an IAM intermediate (Tsavkelova et al., 2012). The orchid endophytic *F. proliferatum* ET1 synthesizes the most IAA among the *Fusarium* examined via the IAM pathway (Tsavkelova et al., 2012). The second pathway involves indole-3-acetaldehyde and is likely responsible for the low level of auxins produced by all other *Fusarium* as this intermediate was detected in mycelium as well as in media of several FFC species (Niehaus et al., 2016). In the smut fungus *Ustilago maydis*, two genes encoding an indole-3-acetaldehyde dehydrogenase and a tryptophan aminotransferase were characterized as responsible for IAA production (Reineke et al., 2008). A possible third pathway for auxins could be mediated by a *Fusarium* gene with homology to the plant gene *YUCCA*, which codes for a key enzyme in plant auxin biosynthesis (Kasahara, 2015) located between the GA and CK clusters. A contribution of the possible YUCCA pathway to auxin production in fungi has not been yet functionally proved.

The phytohormones jasmonates, salicylic acid (SA), ethylene and abscisic acid play a vital role defending plants against fungal pathogens such as *Fusarium oxysporum* (Dempsey and Klessig, 2012; Di et al., 2016). In contrast, the role of CKs and auxins is poorly understood. In some fungus–host interactions, CKs are essential for full virulence of the pathogen (Chanclud et al., 2016; Hinsch et al., 2016). In *Ustilago maydis*, the loss of CK production leads to fewer and smaller tumors in maize (Morrison et al., 2017). The CKs detected in *Magnaporthe oryzae* infected rice leaves have been proposed to serve as a signal to mobilize nutrients, to increase levels of photosynthesis in host leaves or to activate SA-mediated defense responses (Jiang et al., 2013). In *F. mangiferae* infected mango trees, changes in CK and GA levels are associated with the inflorescence and vegetative malformations and reduction in fruit yield (Bist and Ram, 1986; Nicholson and van Staden, 1988).

In addition to changes in GA content in rice seedlings infected by *F. proliferatum*, IAA content was twofold higher in leaves and 1.5-fold lower in roots of a susceptible cultivar. In contrast, changes in a resistant cultivar of the same magnitude were reversed (Quazi et al., 2015). IAA contributes to host vulnerability to a pathogen by inducing acidification and loosening of the cell wall. Resistance to pathogens has been attributed to IAA-amino acid conjugating enzymes which lead to IAA deactivation (Fu et al., 2011). Some bacterial and fungal pathogens can “hijack” auxin metabolism in plant hosts leading to more IAA-aspartic acid (IAA-Asp) conjugate, known to have a role in disease promotion (González-Lamothe et al., 2012). Arabidopsis roots and leaves infected with *F. oxysporum*, and causing wilt disease, show alterations in auxin homeostasis and an up-regulation of plant genes implicated in auxin biosynthesis. However, plant mutants in genes related to auxin synthesis or application of exogenous auxin did not show changes in susceptibility or resistance to this pathogen while mutants defective in auxin signaling and transport conferred pathogen resistance, suggesting a role for auxin in modulating defense responses (Kidd et al., 2011).

In the present study, we examined the relative differences in three major groups of hormones, GAs, auxins and CKs, in plant tissues infected by wild-type (WT) and mutant species of the *F. fujikuroi* complex (FFC). We also examined how FFC species impact expression of maize CK genes during an infection. Finally, the hormonal role in pathogenicity and symptom development (e.g., MMD in mango) is discussed.

## Materials and Methods

### Fungal Strains

*F. fujikuroi* (Ff) IMI58289 (Commonwealth Mycological Institute, Kew, United Kingdom) served as wild type (WT), GA-producing strain. The mango pathogen *F. mangiferae* MRC7560 (Fm), originating in Israel, is deposited in the culture collection of the Medical Research Council (MRC) (Tygerberg, South Africa). *F. proliferatum* NRRL 62905 (*Fp_N*), *F. proliferatum* ET1 (*Fp_E*) and *F. verticillioides* M-3125 (*Fv*) (Fungal Genomics Stock Centre, Kansas State University, FGSC 7600) were provided by Elena Tsavkelova, Moscow State University, Russia, Robert Proctor and Daren W. Brown, United States Department of Agriculture, United States, respectively. The following strains were previously described and were derived from the strains listed above: Ff_IAA, *Fp*_E_IAA, Fm_IAA, Fv_IAA overexpressing both *IAAM*, coding for a tryptophan monooxygenase and *IAAH*, coding for an indole-3 acetamide hydrolase; *Fp_E_IL1, Fm_IL1, Ff_IL1* and *Fv_IL1* overexpressing *IPTLOG1*; *Fp*_*E*_*IL2, Fm_IL2, Ff_IL2* and *Fv_IL2* overexpressing *IPTLOG2*; *Fp_E_IL1P1, Fm_IL1P1, Ff_IL1P1* and *Fv_IL1P1* overexpressing both *IPTLOG1* and *P450-1*; and *Fp_E_IL2P2, Fm_IL2P2, Ff_IL2P2* and *Fv_IL2P2* overexpressing both *IPTLOG2* and *P450-2*. *IPTLOG1/2* and *P450-1/2* originated from *F. fujikuroi* IMI58289 (Table 1) (Niehaus et al., 2016).

**Table 1:** Strains of *Fusarium* species used to infect maize seedlings in the current study and their ability to produce phytohormones in axenic culture.

Strain	Overexpressed genes	Abbreviation	Phytohormone production		
			
			GA	IAA	CK
*F. proliferatum* ET1	–	*Fp_E*	+	+	+
*F. proliferatum* NRRL62905	–	*Fp_N*	LOD	+	+
*F. verticillioides* M-3125	–	*Fv*	LOD	+	+
*F. fujikuroi* IMI58289	–	*Ff*	+++	+	+
*F. mangiferae* MRC7560	–	*Fm*	LOD	+	+
*F. proliferatum* ET1	*FpIAAH, FpIAAM*	*Fp_E_IAA*	ND	+++	+
*F. verticillioides* M-3125	*FpIAAH, FpIAAM*	*Fv_IAA*	ND	+++	+
*F. fujikuroi* IMI58289	*FpIAAH, FpIAAM*	*Ff_IAA*	ND	+++	++
*F. mangiferae* MRC7560	*FpIAAH, FpIAAM*	*Fm_IAA*	ND	+++	+
*F. proliferatum* ET1	*FfIPTLOG1*	*Fp_E_IL1*	ND	ND	+++
*F. proliferatum* ET1	*FfIPTLOG2*	*Fp_E_IL2*	ND	ND	+++
*F. proliferatum* ET1	*FfIPTLOG1, FfP450-1*	*Fp_E_IL1P1*	ND	ND	+++
*F. proliferatum* ET1	*FfIPTLOG2, FfP450-2*	*Fp_E_IL2P2*	ND	ND	+++
*F. verticillioides* M-3125	*FfIPTLOG1*	*Fv_IL1*	ND	+	+++
*F. verticillioides* M-3125	*FfIPTLOG2*	*Fv_IL2*	ND	ND	+++
*F. verticillioides* M-3125	*FfIPTLOG1, FfP450-1*	*Fv_IL1P1*	ND	ND	+++
*F. verticillioides* M-3125	*FfIPTLOG2, FfP450-2*	*Fv_IL2P2*	ND	+	+++
*F. fujikuroi* IMI58289	*FfIPTLOG1*	*Ff_IL1*	ND	ND	+++
*F. fujikuroi* IMI58289	*FfIPTLOG2*	*Ff_IL2*	ND	ND	+++
*F. fujikuroi* IMI58289	*FfIPTLOG1, FfP450-1*	*Ff_IL1P1*	ND	ND	+++
*F. fujikuroi* IMI58289	*FfIPTLOG2, FfP450-2*	*Ff_IL2P2*	ND	ND	+++
*F. mangiferae* MRC7560	*FfIPTLOG1*	*Fm_IL1*	ND	ND	+
*F. mangiferae* MRC7560	*FfIPTLOG2*	*Fm_IL2*	ND	ND	+
*F. mangiferae* MRC7560	*FfIPTLOG1, FfP450-1*	*Fm_IL1P1*	ND	ND	+++
*F. mangiferae* MRC7560	*FfIPTLOG2, FfP450-2*	*Fm_IL2P2*	ND	ND	++


*The number of + characters indicate the levels of hormone detected compared to the respective parent or WT strain; LOD, below the limit of detection; ND, not determined.*

### Generation of New Fungal Strains

Creation of deletion and overexpression vectors was accomplished via a yeast recombinational cloning system using *Saccharomyces cerevisiae* strain FGSC 9721 (FY834) obtained from the Fungal Genetics Stock Center, Kansas State University (Schumacher, 2012). The deletion vectors contained about 1 kb of the 5′ flank and 3′ flank of the respective target gene that were amplified with 5F/5R and 3F/3R primers, respectively (Supplementary Table S1). The hygromycin resistance cassette was amplified from pSCN44 as template with the primer pair Hph-F/Hph-R. The cassette consists of the hygromycin B phosphotransferase gene (*hph*) and the *trpC* promoter from *Aspergillus nidulans* (Staben et al., 1989). The resistance cassette, the shuttle vector pRS426 (Christianson et al., 1992) and the 5′ and 3′ flanks were cloned into FY834 creating vectors pΔ*Fm_IL1* and pΔ*Fm_tI*. For deletion of *IPTLOG1* in the Δ*Fm_tI* background, a nourseothricin resistance gene driven by the *oliC* promoter from *A. nidulans* was used. To overexpress (OE) *IPTLOG1* and *IPTLOG2* in *F. mangiferae*, the respective genes were amplified by PCR from *F. mangiferae* genomic DNA. The amplified genes together with a *Not*I and *Nco*I restricted plasmid pNAN-OGG (Schumacher, 2012), containing a hygromycin resistance cassette, were transformed into FY834 creating vectors pOE:*FmIL1* and pOE:*FmIL2*. The PCR derived fragments were verified by sequence analysis using the BigDye^®^Terminator v3.1 cycle sequencing kit and the ABI Prism^®^3730 Genetic Analyzed (Applied Biosystems, Foster City, CA, United States).

Transformation of *Fusarium* spp. was carried out according to Wiemann et al. (2013). Regeneration of transformed protoplasts was performed over 4–5 days at 28°C in regeneration medium (0.7M sucrose, 0.05% yeast extract) containing either 100 μg.mL^-1^ nourseothricin (Werner-Bioagents, Germany) or 100 μg.mL^-1^ hygromycin (Calbiochem, Germany). Transformants were purified by single spore isolation to homokaryons. Vector integration events were confirmed by diagnostic PCR using appropriate primers (Supplementary Table S1). For this work, the following *F. mangiferae* mutants were generated: Δ*Fm_IL1* (deletion of *IPTLOG1*), Δ*Fm_tl* (deletion of *tRNA-IPT*), ΔΔ*Fm_IL1/tl* (deletion of both *IPTLOG1* and *tRNA-IPT*), *Fm_FmIL1* (over-expression of Fm*IPTLOG1*), *Fm_FmIL2* (over-expression of Fm*IPTLOG2*) and *Fm_FjIL1* (over-expression of Ff*IPTLOG1*).

### Fungal Cultivation Methods

Conidial inoculum of each *Fusarium* strain, used for maize seedling infections in the hydroponic system, were prepared from 7-day-old potato dextrose agar plates, cultivated at 28°C. Spores were mechanically dislodged with a loop in 10 mL of sterile water, filtered and the spore concentration was determined using a Bürker counting chamber. Conidial inoculum for the maize seedling assay in soil were prepared by growing *Fusarium* strains for 3 days in mung bean medium at 28°C (Bai and Shaner, 1996).

For CK quantification, *F. mangiferae* strains were first cultivated for 3 days in 300-mL Erlenmeyer flasks with 100 mL Darken medium (Darken et al., 1959) on a rotary shaker at 180 rpm at 28°C; 500 μL of this culture was then used to inoculate 100 mL of ICI (Imperial Chemical Industries, United Kingdom) media (Geissman et al., 1966) containing 60 mM glutamine as a nitrogen source and 40 g per L of glucose. Growth proceeded for 7 days on a rotary shaker at 28°C in the dark. The culture filtrates were harvested and lyophilized prior to analysis.

### Plant Material

Hybrid white sweet maize variety Silver Queen (Johnnyseeds, United States) was used for all maize related experiments. Roots and shoots of infected maize seedlings were separated and lyophilized before hormone profiling and gene expression analysis. Infected mango material was obtained from a 25-year-old heavily infected mango malformation diseased orchard (cv. Keitt) located in northern Israel, close to Kibbutz Ma’agan (320 42’ 23″ N; 36’ 31″ E). Healthy mango material was obtained from 3-year-old trees (cv. Keitt) cultivated in a nursery located in the Volcani Center, Bet Dagan, Israel. The various sampled tissues were as follows: diseased malformed and healthy inflorescence tissue (young panicles, 1-month after development), diseased malformed and healthy inflorescence tissue (mature panicles, 2- to 3-month after development), swollen and healthy buds (1-month before bud break). Immediately after sampling, floral and bud material was frozen in liquid nitrogen and lyophilized.

### Virulence Assays

#### Maize Seedling Assay in Soil

Maize seeds were sterilized by soaking in 0.82% sodium hypochlorite for 1 min and then rinsed twice in sterile water for 1 min. The seeds were inoculated by soaking for 2 days in 30 mL of mung bean medium cultures of individual *Fusarium* strains. Ten seeds were sown in a water-saturated soil mixture, consisting of sphagnum peat moss, vermiculite and dolomite lime (Sunshine Redi-Earth Professional Growing Mix), in a 10-cm^2^ plastic pot to a depth of 1 cm. The pots were incubated in a growth chamber with a light dark cycle consisting of 14 h light at 30°C and 10 h dark at 20°C.

Disease severity was assessed as follows: (i) at 7 days after sowing, percent germination was determined by counting the number of seedlings per 10 seeds sown in each pot; (ii) at 20 days after sowing, seedling height was determined by measuring the height of each seedling from the soil line to the top of the longest leaf; and (iii) seedling weight was determined as fresh weight by cutting a seedling at the soil line. The resulting data were subjected to analysis of variance (ANOVA), and statistically significant differences between means were determined by a least squares means test using a Bonferroni adjustment. These analyses were done using SAS Statistical Software (SAS Institute Inc.).

#### Maize Seedling Assay in Hydroponic System

Maize seeds were sterilized by first soaking in 4% sodium hypochlorite for 10 min, rinsed twice with sterile water, and then soaking in 70% ethanol for 1 min and rinsed twice with sterile water. Seeds were inoculated by soaking in water containing 10^6^ *Fusarium* spores per mL in a flask on a rotary shaker overnight (120 rpm). Inoculated seeds were dried for 2 h and placed in a petri dish with moistened filter paper to germinate in the dark at 26°C. After 2 days, germinated seeds were moved into a hydroponic system consisting of plastic boxes with Hoagland solution. Seedlings were incubated in a growth chamber with a light/dark cycle (16 h/8 h) at the constant temperature of 25°C.

### Plant Hormone Extraction and Quantification

Control and infected maize seedling shoots and roots were analyzed for GAs using the method described by Urbanová et al. (2013) with minor modifications. Briefly, approximately 10 mg of lyophilized tissue were ground to a powder using 3-mm zirconium oxide beads and extracted overnight at 4°C with 1 mL of ice-cold 80% acetonitrile containing 5% formic acid. Seventeen internal GAs standards ([^2^H_2_]GA_1_, [^2^H_2_]GA_3_, [^2^H_2_]GA_4_, [^2^H_2_]GA_5_, [^2^H_2_]GA_6_, [^2^H_2_]GA_7_, [^2^H_2_]GA_8_, [^2^H_2_]GA_9_, [^2^H_2_]GA_15_, [^2^H_2_]GA_19_, [^2^H_2_]GA_20_, [^2^H_2_]GA_24_, [^2^H_2_]GA_29_, [^2^H_2_]GA_34_, [^2^H_2_]GA_44_, [^2^H_2_]GA_51_ and [^2^H_2_]GA_53_; purchased from professor Lewis Mander, Australia) were added to each sample. The homogenates were centrifuged at 19,000 rpm at 4°C for 10 min, and the resulting supernatants were passed through an ion exchange SPE cartridges (Waters) prior to analysis by high pressure-liquid chromatography-tandem mass spectrometry (Micromass). GAs were detected using multiple-reaction monitoring mode of the transition of the ion [M–H]^-^ to the appropriate product ion. The Masslynx 4.1 software (Waters) was used to quantify the GAs levels by the standard isotope dilution method (Rittenberg and Foster, 1940).

Levels of the auxin IAA and IAA metabolites were determined in the maize seedling shoots and roots using the method described by Novák et al. (2012). Briefly, approximately 5 mg of lyophilized tissue was extracted with 1 mL cold phosphate buffer (50 mM; pH 7.0) containing 0.1% sodium diethyldithiocarbamate, supplemented with internal standards. After centrifugation at 20,000 rpm for 10 min, one half of each sample was acidified with 1 M HCl to pH 2.7 and subjected to solid phase extraction using an Oasis^TM^ HLB column (Waters). For quantification of indole-3-pyruvic acid, the second half of the sample was derivatized with cysteamine (0.25 M, pH 8.0) for 1 h, acidified with 3 M HCl to pH 2.7 and purified by solid phase extraction. After evaporation under reduced pressure, samples were analyzed for auxin content by Acquity UPLC^TM^ linked to Xevo TQ MSTM (Waters).

Levels of CKs were determined in the maize seedling shoots and roots essentially as described by Hinsch et al. (2015) using a high-pressure liquid chromatography (Acquity UPLC^TM^; Waters) coupled to a triple quadrupole mass detector (Xevo TQ MSTM; Waters) equipped with an electro-spray interface. To check CK recovery and to validate peak identity, isotope-labeled CK internal standards (OlChemIm, Czechia) were added, each at 1 pmol, to samples prior to extraction. Levels of CKs were determined in lyophilized culture filtrates of *F. mangiferae* without addition of internal standards. Filtrates were purified on immuno-affinity columns (OlchemIm) after pre-purification on Speed SPE Octadecyl C18 cartridges (Applied Separation) and Oasis MCX cartridges (Waters) as described by Hinsch et al. (2016). CKs were quantified after separation on a C18 reverse-phase column (ZORBAX RRHD Eclipse Plus 1.8 μm, 2.1 × 150 mm, Agilent) coupled to the Ultra performance liquid chromatography (Shimadzu Nexera).

Cytokinin dehydrogenase (CKX) activity was measured in extracts of lyophilized and powdered maize seedling shoots and roots extracted with 20-fold excess (w/v) of 0.2M Tris/HCl, pH 8.0, 0.3% Triton-X. The CKX activity was determined spectrophotometrically with 0.5 mM dichlorophenolindophenol as an electron acceptor and 0.25 mM isopentenyladenine as a substrate (Frébort et al., 2002). All measurements were performed in four biological replicates. The protein content was estimated by the method of Bradford (1976) with bovine serum albumin as a standard.

All results are presented as mean value ± standard deviation from at least three independent biological replicates; statistical significance of results was revealed by Student’s unpaired *t*-tests or ANOVA at *p* ≤ 0.05 (Statistica 13.3, TIBCO Software Inc.).

### Gene Expression Profiling by Quantitative Real-Time PCR (qPCR)

Total RNA was isolated from roots and shoots of maize seedlings grown in a hydroponic system and infected by wild-type strains of *F. mangiferae, F. verticillioides* and *F. fujikuroi*. Each treatment was analyzed in three independent biological replicates; each biological replicate was represented by the shoots or roots of five maize seedlings. Approximately 100 mg of tissue for each independent biological replica (*n* = 3) was ground in liquid nitrogen and RNA was extracted and purified with the RNAqueous kit (Thermo Fisher Scientific). The RNA was treated with DNAse (TURBO DNA-free kit; Thermo Fisher Scientific) and cDNA was obtained using a RevertAid First Strand cDNA Synthesis Kit (Thermo Fisher Scientific) from 2 μg of total RNA as the template, according to the manufacturer’s instructions. qPCR was performed using TaqMan Gene Expression Master Mix (Thermo Fisher Scientific) in a Viia7^TM^ Real-Time PCR System (Thermo Fisher Scientific). For both fungal and maize genes, primers and TaqMan probes were designed with Primer Express 3.0 software (Thermo Fisher Scientific, Supplementary Table S1). The maize target genes used here were identified in a previous study (Vyroubalová et al., 2009). For each condition, the three independent biological replicates were analyzed in three technical replicates. Expression of fungal genes was measured by absolute quantification, whereas maize gene expression was obtained by relative quantification according to ΔΔCt method (Schmittgen and Livak, 2008). To ensure that primers amplified the desired gene target, amplicons for every primer pair were cloned into the pDRIVE vector (Qiagen) and sequenced. The cloned PCR products were also used as template to determine PCR efficiency and absolute levels of gene transcript in isolated RNA. The relative expression of the maize genes were normalized with respect to β*-*actin (BT086225) and elongation factor 1 (AF136829.1) gene expression. Expression values were determined and statistically evaluated with DataAssist v3.0 Software (Thermo Fisher Scientific).

## Results

**FIGURE 1:**
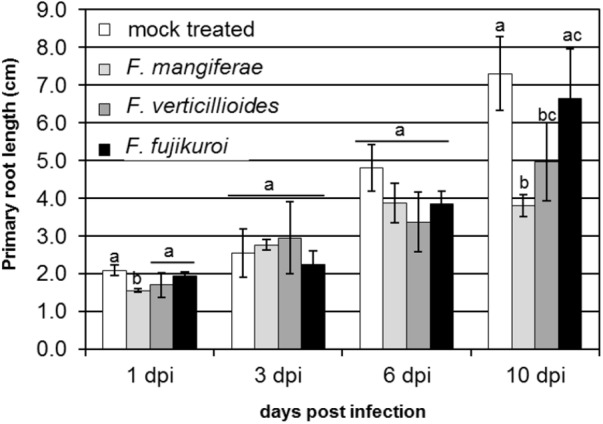
Effect of infection by *F. verticillioides, F. mangiferae* and *F. fujikuroi* on the length of primary root of maize seedlings. Hydroponic growth of seedlings for 10 days. Average value of 20 infected seedlings with standard deviations is presented. Bars indicated by the same letter are not statistically significantly different (ANOVA; *p* ≤ 0.1).

We previously demonstrated that *F. proliferatum* strain ET1 (isolated from the roots of an epiphytic orchid), *F. fujikuroi* (a pathogen of rice) and *F. mangiferae* (a pathogen of mango) are able to penetrate and invade maize seedlings and to cause blight disease symptoms similar to *F. verticillioides* and *F. proliferatum* strain NRRL 62905, two pathogens of maize (Niehaus et al., 2016). Here, we found that *F. mangiferae* also can cause seedling disease by affecting the growth of maize primary roots and overall root system development (Figure 1). In the current study, we examined the hormonal status in maize plant tissues infected by the following *Fusarium*: *F. verticillioides, F. proliferatum* strain NRRL 62905, *F. proliferatum* strain ET1, *F. fujikuroi* and *F. mangiferae*, as well as auxin/CK accumulating or deficient mutant strains derived thereof. All of the strains were able to synthesize CK in axenic culture while mutants overexpression *IPTLOG1* and *P450-1* accumulated more (Table 1). *F. proliferatum* ET1, *F. verticillioides, F. fujikuroi* and *F. mangiferae* strains expressing *IAAH* and *IAAM* from *F. proliferatum* synthesized more IAA than wild-type (Table 1). Some GAs were detected in *F. proliferatum* while significantly more was detected in *F. fujikuroi* axenic cultures (Table 1).

### Infection by *Fusarium* Induced Changes in Gibberellins (GAs) Status in Maize Seedlings

Gibberellin content was measured in infected roots and shoots 10 days post inoculation (dpi) as active forms (GA_1_, GA_3_, GA_4_, GA_5_, GA_7_, GA_13_), precursors (GA_9_, GA_12_, GA_15_, GA_19_, GA_20_, GA_24_, GA_44_, and GA_53_) and deactivation products (GA_8_, GA_29_, GA_34_, and GA_51_) (referred to as GA turnover) (Table 2). 13-Hydroxylated (GA_1_, GA_3_, GA_5_) and 13-non-hydroxylated (GA_4_, GA_7_, GA_13_) active forms, precursors, and deactivation products were also measured (Supplementary Table S2).

**Table 2:** Quantification of gibberellins (GAs) in maize seedlings grown in soil and infected by different *Fusarium* strains (10 dpi).

	Mock-treated	*Fp_N*	*Fp_E*	*Fm*	*Ff*	*Fv*
	**Roots of infected plants**					

**Active GAs**	26.8 ± 1.1	25.5 ± 3.4	75.3 ± 22.2^∗^	36.2 ± 1.9^∗^	11776 ± 665.6^∗^	20.5 ± 1.3^∗^
**Precursors**	367.0 ± 81.1	619.7 ± 72.4^∗^	843.2 ± 100.5^∗^	928.2 ± 59.7^∗^	1159.6 ± 59.2^∗^	492.5 ± 35.2^∗^
**Turnover**	233.4 ± 40.2	373.1 ± 14.6^∗^	437.4 ± 28.0^∗^	726.8 ± 81.2^∗^	12134.0 ± 649.7^∗^	209.8 ± 9.8

	**Shoots of infected plants**					

**Active GAs**	27.4 ± 0.4	71.4 ± 7.7^∗^	35.2 ± 9.5^∗^	30.1 ± 2.4	325.2 ± 33.2^∗^	15.7 ± 1.5^∗^
**Precursors**	636.1 ± 61.4	625.7 ± 75.9	484.9 ± 56.1^∗^	530.8 ± 45.2	634.7 ± 86.7	339.9 ± 27.0^∗^
**Turnover**	180.0 ± 21.5	304.4 ± 20.5^∗^	143.5 ± 26.6^∗^	108.9 ± 7.4^∗^	552.2 ± 67.6^∗^	194.0 ± 2.0


*Mean values with standard deviations obtained from four to six biological replicates (independent seedlings) are presented; ^∗^ indicates significant differences between mock and *Fusarium*-infected tissue according to Student’s unpaired *t*-tests at *p* ≤ 0.05 (*n* = 4–6). Concentrations of phytohormones are in pmol per g dry weight plant material. Active GAs: sum of active GAs (GA_1_, GA_3_, GA_4_, GA_5_, GA_7_, GA_13_); precursors: sum of GA precursors (GA_9_, GA_12_, GA_15_, GA_19_, GA_20_, GA_24_, GA_44_, and GA_53_); turnover: sum of deactivated GAs (GA8, GA29, GA34, and GA51); *Fp_N, F. proliferatum* NRRL62905; *Fp_E, F. proliferatum* ET1; *Fm, F. mangiferae*; *Ff, F. fujikuroi*; *Fv, F. verticillioides*.*

In the roots of maize seedlings infected by *F. verticillioides* and *F. proliferatum* NRRL62905, a slight decrease or no change in active GAs was observed. This, coupled with an increase in GA precursor and deactivated product, suggest a tight regulation of GA status in the roots upon infection. In contrast, a marginal to significant increase in active GAs was observed in the root of maize infected by *F. mangiferae* (3-fold), *F. proliferatum* ET1 (1.9-fold) and *F. fujikuroi* (52-fold) (Table 2). The accumulation of active GAs was consistent with the accumulation of GA precursors, notably the 13-non-hydroxylated GA_9_ and GA_12_, observed in the maize roots infected by all of the *Fusarium* species (Supplementary Table S2). Because these three species also produce significant GAs in axenic culture, the increased GAs we detected in infected plants might reflect a failure in the ability of the plant to maintain root GA homeostasis upon infection.

The amount of GAs detected in shoots of mock-treated maize seedlings was similar to the amount detected in roots. Active GAs accumulated significantly more in the shoots of maize seedlings whose roots were infected only by *F. proliferatum* NRRL 62905 (native maize pathogen) and *F. fujikuroi*. In contrast to roots, active GA accumulation in the shoots appeared related to an accumulation of GA_12_ (13-non-hydroxylated) and GA_53_ (13-hydroxylated). Also GA_9_ levels in infected shoots were twofold to fourfold lower than those found in the non-infected shoots. Taken together, these results suggest that the root-to-shoot translocation of maize-generated GAs was reduced during the infection process, or that the biosynthesis or origin of shoot GAs is via a different mechanism (Supplementary Table S2). Interestingly the infection by *F. verticillioides*, the other native maize pathogen, led to a decrease in active GAs in both roots and shoots of infected seedlings, suggesting that success of infection of maize by *F. verticillioides* might be independent of GAs, at least 10 days post inoculation.

### Infection by *Fusarium* Induced Changes in Auxin (IAA) Status in Maize Seedlings

The levels of auxin IAA, its precursors and deactivation products were analyzed in the roots of maize seedlings 10 days after infection with different *Fusarium* species (Table 3 and Supplementary Table S3). Infection by any of the fusaria (except *F. fujikuroi*) seemingly induced the accumulation of free IAA although only significant for *F. mangiferae* (Table 3). The amount of IAA-glucose, a storage form of IAA, was significantly reduced in the infected roots of maize seedlings in four of the five fusaria. It is possible that the accumulation of free IAA resulted from the release of IAA from the glucose-conjugate. No significant difference in IAA precursor content could be observed between mock-treated roots and infected roots. Overall, infection led to an accumulation of compounds related to IAA degradation, especially IAA-Asp which may be indicative of IAA turnover needed to maintain basal endogenous IAA levels (Supplementary Table S3). Albeit not significant, we did note an increase in the indole-3-pyruvic acid (IPyA), an IAA precursor of the YUCCA pathway, in tissue infected with *F. mangiferae*, suggesting the activation of this pathway in either the plant or fungus. No significant difference in the pool of auxin and auxin derivatives between the mock-treated and infected seedlings was noted, except for *F. mangiferae* (Supplementary Table S3) which suggests that most *Fusarium* strains did not secrete substantial amounts of auxins into the plant tissue and that the auxins that were detected were likely of plant origin.

**Table 3:** Quantification of auxin (IAA) in the roots of maize seedlings grown in soil and infected by different *Fusarium* strains (10 dpi).

	Mock-treated	*Fv*	*Fp*_N	*Fp*_E	*Fm*	*Ff*	*Fp*_E_IAA	*Ff*_IAA
Active IAA	590 ± 106	1501 ± 958	1004 ± 421	1520 ± 618	3723 ± 848^∗^	473 ± 88	21234 ± 19574	57402 ± 24206^∗^
Total precursors	6782 ± 2041	7435 ± 707	6188 ± 2595	10820 ± 3955	13158 ± 4700^∗^	4784 ± 3508	35152 ± 16119^∗^	77237 ± 34802^∗^
Storage (IAA-Glc)	4833 ± 1603	261 ± 33^∗^	593 ± 0	574 ± 37^∗^	239 ± 68^∗^	888 ± 126^∗^	n.d.	n.d.
Total turnover	11267 ± 2582	9136 ± 2220	6799 ± 1304^∗^	7047 ± 766^∗^	9734 ± 1526^∗^	5093 ± 876^∗^	32401 ± 25657	75963 ± 20478^∗^


*Mean values with standard deviations obtained from three biological replicates (independent seedlings) are presented; ^∗^ indicates significant differences between mock-treated and Fusarium-infected tissue according to Student’s unpaired t-tests at p ≤ 0.05 (n = 3). Concentrations of phytohormones are in pmol per g dry weight plant material. Fv, F. verticillioides; Fp_N, F. proliferatum NRRL62905; Fp_E, F. proliferatum ET1; Fm, F. mangiferae; Ff, F. fujikuroi; Fp_E_IAA, Fp_E strain overexpressing IAAM and IAAH genes; Ff_IAA, F.f strain overexpressing IAAM and IAAH genes; IAA, indole-3-acetic acid; IAA-Glc, IAA-glucose; n.d., not determined.*

**FIGURE 2:**
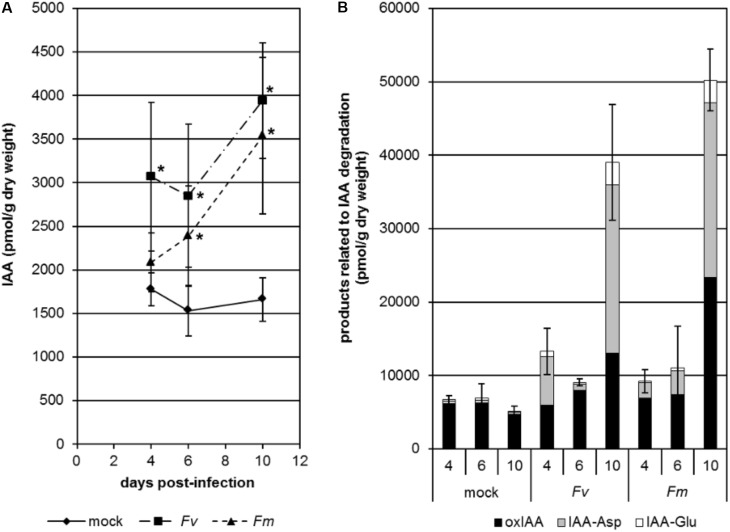
Changes in free active IAA **(A)** and products related to IAA degradation **(B)** in the roots of maize seedlings infected by *F. verticillioides* (*Fv*) and *F. mangiferae* (*Fm*). The analysis was performed after 4, 6, and 10 days of hydroponic growth post inoculation (dpi). Graphs represent the mean value of three independent biological replicates; bars represent the standard error. ^∗^ indicates significant differences between mock and *Fusarium*-infected tissue according to Student’s unpaired *t*-tests at *p* ≤ 0.05 (*n* = 3). IAA, indole-3-acetic acid; oxIAA, 2-oxindole-3-acetic acid; IAA-Asp, IAA-aspartate; IAA-Glu, IAA-glutamate.

In order to study the dynamics of IAA changes during infection by *Fusarium*, we analyzed the content of free IAA and products related to IAA degradation in the roots of maize seedlings infected with *F. verticillioides* or *F. mangiferae* grown hydroponically (Figure 2). In both cases, we saw an accumulation of free IAA in the roots as soon as 4 dpi (Figure 2A). At the end of the experiment, i.e., at 10 dpi, free IAA was significantly increased more than twofold in fungal infected seedlings. Accumulation of compounds related to IAA turnover was also observed during infection. This was particularly significant at 10 dpi with IAA-Asp contributing to the greatest extent (Figure 2B).

### Infection by *Fusarium* Affects CK Status in Maize Seedlings

The four plant CKs were extracted and quantified from roots and shoots of maize seedlings infected with different *Fusarium* species grown in soil for 10 days (Table 4 and Supplementary Table S4). Infection by both *F. proliferatum* species did not significantly modify the overall CK levels while infection by *F. verticillioides* and *F. fujikuroi* led to a modest decrease in CK levels (1.4- and 2.2-fold, respectively) in shoots only. In contrast, infection by *F. mangiferae* led to a modest increase in CK levels (1.4-fold) in shoots only. We did note that the predominant CK present in the seedlings was *c*Z which accounted for most of the overall change in total CKs observed (Table 4 and Supplementary Table S4). The overall increase in free active CK correlated with a concomitant increase in CK precursors and glucoside derivatives suggesting that mechanisms exist to adjust levels of endogenous CKs within the plant due to changing growth conditions (Supplementary Table S4). The most significant increases observed were for tissues infected with *F. mangiferae* which contained significantly more CKs of all types, except for *c*Z derivatives. In roots, the amount of the two major active CKs, iP and *t*Z, were 4- and 53-fold higher, respectively; in the shoots, iP and *t*Z was 5- and 11-fold higher than observed in mock-treated plants, respectively (Supplementary Table S4). Infection by *F. fujikuroi* did not affect CK status in the roots. Our observation that maize seedlings infected with *F. fujikuroi* overexpressing CK biosynthetic genes *FfIPTLOG2* and *FfP450-2* (*Ff_IL2P2*) accumulated even more iP, *t*Z and DHZ suggest that the fungi were able to produce CKs *in planta* (Table 4 and Supplementary Table S4).

**Table 4:** Quantification of maize cytokinins and CKX activity in seedlings grown in soil and infected by different *Fusarium* strains (10 dpi).

	Mock-treated	*Fv*	*Fp_N*	*Fp_E*	*Fm*	*Ff*	*Ff_IL2P2*
	***Roots of infected plants***						

Total iP	319.6 ± 36.1	414.2 ± 27.3^∗^	328.7 ± 72.6	239.4 ± 68.8	655.0 ± 160.8^∗^	317.4 ± 55.6	1038.7 ± 318.5^∗^
Total *t*Z	215.1 ± 28.2	303.3 ± 55.8	189.0 ± 46.3	171.4 ± 52.8	1017.7 ± 275.7^∗^	209.6 ± 62.7	4276.9 ± 196.7^∗^
Total *c*Z	5276.2 ± 783.5	6241.8 ± 1188.6	5976.7 ± 1215.2	5424.1 ± 765.6	7730.9 ± 1555.8	5425.0 ± 332.4	6738.4 ± 934.7
Total DHZ	35.8 ± 2.5	58.0 ± 4.4^∗^	35.4 ± 9.5	41.5 ± 11.2	571.6 ± 89.1^∗^	32.9 ± 9.1	1527.0 ± 340.6^∗^
Total CKs	5846.7 ± 735.7	7017.3 ± 1120.4	6288.0 ± 1177.6	5876.4 ± 887.2	9975.2 ± 1658.9^∗^	5984.8 ± 438.8	13581.1 ± 711.3^∗^
CKX activity	0.54 ± 0.32	1.37 ± 0.57	1.60 ± 0.65	0.99 ± 0.42	147 ± 50^∗^	1.39 ± 0.55	16.3 ± 8.32^∗^

	***Shoots of infected plants***						

Total iP	31.8 ± 7.7	56.7 ± 15.9	167.0 ± 50.1^∗^	112.0 ± 28.4^∗^	176.8 ± 30.9^∗^	151.6 ± 41.7^∗^	n.d.
Total *t*Z	17.2 ± 1.6	29.7 ± 5.1^∗^	16.2 ± 0.2	20.6 ± 0.2	555.6 ± 105.7^∗^	21.4 ± 5.6	n.d.
Total *c*Z	5031.0 ± 980.8	3482.1 ± 103.4^∗^	4303.4 ± 227.3	4214.4 ± 578.8	9110.9 ± 1034.7^∗^	2327.1 ± 293.0^∗^	n.d.
Total DHZ	39.3 ± 10.2	17.4 ± 1.7^∗^	32.3 ± 7.7	49.1 ± 5.8	2721.5 ± 630.1^∗^	45.3 ± 9.0	n.d.
Total CKs	5119.2 ± 972.6	3559.6 ± 95.3^∗^	4518.9 ± 199.3	4396.2 ± 555.7	12564.7 ± 486.7^∗^	2545.5 ± 339.0^∗^	n.d.
CKX activity	0.21 ± 0.10	4.11 ± 2.34^∗^	0.52 ± 0.33	0.93 ± 0.45	12.3 ± 7.86^∗^	0.87 ± 0.36	n.d.


*Mean values with standard deviations obtained from three biological replicates (independent seedlings) are presented; ^∗^ indicates significant differences between mock and *Fusarium*-infected tissue according to Student’s unpaired *t*-tests at *p* ≤ 0.05 (*n* = 3). Concentrations of phytohormones are in pmol per g dry weight plant material. The cytokinin oxidase/dehydrogenase (CKX) activity is expressed as pkat per mg of extracted proteins; iP, isopentenyladenine; *t*Z, *trans-*zeatin; *c*Z, *cis*-zeatin; DHZ, dihydro-zeatin; *Fp_N, F. proliferatum* NRRL62905; *Fp_E, F. proliferatum* ET1; *Fm, F. mangiferae*; *Ff, F. fujikuroi*; *Fv, F. verticillioides*; *Ff*_IAA, *F. fujikuroi* overexpressing *IAAM* and *IAAH* genes; *Ff*_IL2P2, *F. fujikuroi* overexpressing *IPTLOG2* and *P450-2* genes; ND, not determined.*

Cytokinin levels *in planta* are regulated *inter alia* by irreversible degradation by cytokinin oxidase/dehydrogenase (CKX). Therefore, we measured CKX activity in the roots and shoots of maize seedlings infected by the different *Fusarium*. CKX activity was most strongly stimulated in the roots and shoots infected with *F. mangiferae* and the CK-overproducing strain *Fj_IL2P2*. A small but significant increase in CKX activity was also observed in the shoots, but not roots, infected by *F. verticillioides* which was consistent with an overall decrease in CK content compared to shoots of mock-treated seedlings (Table 4). Taken together, these data suggest that the regulation of CK levels upon infection by *Fusarium* involves production and glucosylation as well as degradation by CKX.

**FIGURE 3:**
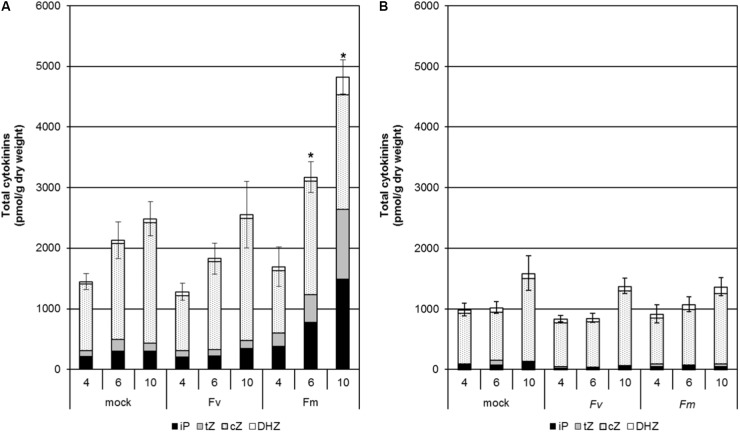
Changes in content of the different active CKs in the roots **(A)** and shoots **(B)** of maize seedlings infected with *F. verticillioides* and *F. mangiferae*. The analysis was performed at 4, 6, and 10 days of hydroponic growth post inoculation (dpi). Graphs represent the mean value of three independent biological replicates, each containing a pool of five seedlings; bars represent the standard error. ^∗^ indicates significant differences between mock and *Fusarium*-infected tissue according to Student’s unpaired *t*-tests at *p* ≤ 0.05 (*n* = 3). Concentrations of hormones are in pmol per g dry weight. The sums of all isopentenyladenine (iP), *trans-*zeatin (*t*Z), *cis-*zeatin (*c*Z) and dihydrozeatin (DHZ) type cytokinins are presented.

We also determined CK levels in maize seedlings grown hydroponically during infection by *F. verticillioides* and *F. mangiferae* at 4, 6, and 10 dpi (Figure 3). An increase in total CK content was observed in the roots of mock-treated seedlings over the time course indicating that CKs naturally accumulate during development (Figure 3A). The same trend was observed for roots of seedlings infected by both *Fusarium*. The observation that roots infected by *F. mangiferae* accumulated significantly more CKs than the non-infected roots suggest that this fungus either induced additional plant CK synthesis, or produced CKs itself which then accumulated in the roots. The same observation was noted during the interaction between *F. mangiferae* and mango, its natural host (Supplementary Table S5). In contrast, the overall CK content was lower in shoots of maize seedlings infected by fusaria than in the roots. No significant change in CK content was observed during the time-course, either upon infection by *F. verticillioides* or *F. mangiferae* (Figure 3B).

### Expression of Fungal and Plant Genes Involved in CK Metabolism During *Fusarium*–Maize Interaction

To study the origin of CK accumulation in *F. mangiferae-*infected maize *tissues*, the expression of fungal genes encoding enzymes involved in CK synthesis (*IPTLOG1* and *IPTLOG2*), as well as maize genes encoding enzymes involved in CK metabolism (*IPT* and *CKX*) were examined over 10 days post inoculation by qPCR (Tables 5, 6). Total RNA was extracted from roots and shoots of seedlings infected by *F. mangiferae, F. verticillioides* and *F. fujikuroi*. The abundance of fungal transcripts coding for ubiquitin (*FmUBI*) and actin (*FvACT* and *FfACT*), two common housekeeping genes, served to follow the growth of the fungi (Table 5). The increase in transcripts in infected roots observed over time strongly indicate that all three fungi were able to colonize the plant roots and thrive. Fungal gene transcripts were detected in the shoots of maize only at 4 dpi and later. Of the fungal CK synthesis genes, *IPTLOG2* transcripts were much more readily and consistently present in roots infected with all three fungi (Table 5). While transcript for *FmIPTLOG2* increased steadily with a maximum at 10 dpi, transcripts for *FvIPTLOG2* peaked at 6 dpi, fourfold higher than *FmIPTLOG2* at 10 dpi. In contrast, expression of *FfIPTLOG2* was low at 3–4 dpi and decreased significantly out to 10 dpi. In shoots, fungal *IPTLOG2* transcripts were detectable only after infection by *F. verticillioides* (Table 5).

**Table 5:** Expression of fungal *IPTLOG* genes in the roots and shoots of maize seedlings infected by *F. mangiferae, F. verticillioides*, and *F. fujikuroi.*

In the roots of maize seedlings						
	**Genes**	**1 dpi**	**3 dpi**	**4 dpi**	**6 dpi**	**10 dpi**

*F. mangiferae*	*FmUBI*	19.1 ± 7.32	889 ± 492	2169 ± 823	2545 ± 285	7854 ± 3600
	*FmIPTLOG1*	n.d.	n.d.	n.d.	0.96 ± 0.57	0.35 ± 0.26
	*FmIPTLOG2*	n.d.	0.85 ± 0.74	17.4 ± 7.30	88.9 ± 58.6	716 ± 494
*F. verticillioides*	*FvACT*	7570 ± 1738	222859 ± 114805	450178 ± 157031	281578 ± 110834	232959 ± 13051
	*FvIPTLOG1*	n.d.	n.d.	2.86 ± 2.48	0.44 ± 0.16	n.d.
	*FvIPTLOG2*	n.d.	114 ± 61.3	1381 ± 599	2868 ± 1997	1121 ± 228
*F. fujikuroi*	*FfACT*	214 ± 108	18608 ± 10042	15222 ± 2830	57216 ± 18166	85585 ± 36355
	*FfIPTLOG1*	n.d.	0.47 ± 0.20	5.85 ± 2.34	n.d.	0.26 ± 0.22
	*FfIPTLOG2*	7.70 ± 5.25	106 ± 48.2	73.5 ± 31.6	n.d.	3.97 ± 2.09

**In the shoots of maize seedlings**						

	**Genes**	**1 dpi**	**3 dpi**	**4 dpi**	**6 dpi**	**10 dpi**

*F. mangiferae*	*FmUBI*	–	–	121 ± 40.8	467 ± 214	183 ± 34.1
	*FmIPTLOG1*	–	–	n.d.	1.10 ± 1.55	n.d.
	*FmIPTLOG2*	–	–	1.83 ± 1.02	n.d.	n.d.
*F. verticillioides*	*FvACT*	–	–	16175 ± 8006	78167 ± 36366	106034 ± 52104
	*FvIPTLOG1*	–	–	n.d.	2.87 ± 2.41	n.d.
	*FvIPTLOG2*	–	–	344 ± 485	121 ± 143	n.d.
*F. fujikuroi*	*FfACT*	–	–	1872 ± 920	14695 ± 8425	18350 ± 3175
	*FfIPTLOG1*	–	–	0.03 ± 0.05	n.d.	n.d.
	*FfIPTLOG2*	–	–	n.d.	n.d.	n.d.


*Quantity of *IPTLOG1* and *IPTLOG2* transcripts and transcripts for the house-keeping genes *UBI* (*F. mangiferae*) and ACT (*F. verticillioides* and *F. fujikuroi*) in 1ng of total RNA extracted from the root and the shoot of infected plants. Each value is mean presented with standard deviation of six biological replicates (seedlings). ^∗^Significant difference between mock and *Fusarium*-infected tissue according to Student’s unpaired *t*-tests at *p* ≤ 0.05. n.d., not detected; –, not analyzed.*

**Table 6:** Expression of maize Zm*CKX1* and Zm*IPT* genes in the roots and shoots of seedlings infected by *F. mangiferae, F. verticillioides* and *F. fujikuroi.*

In the roots of maize seedlings						
	**Genes**	**1 dpi**	**3 dpi**	**4 dpi**	**6 dpi**	**10 dpi**

***F. mangiferae***	*ZmIPT5*	0.72 ± 0.23	3.34 ± 0.42^∗^	0.97 ± 0.22	0.67 ± 0.13^∗^	1.45 ± 0.37
	*ZmIPT3b*	0.88 ± 0.26	2.48 ± 0.94	1.82 ± 0.63	0.70 ± 0.13^∗^	0.57 ± 0.04^∗^
	*ZmIPT6*	1.10 ± 0.43	13.9 ± 4.43^∗^	1.02 ± 0.05	0.56 ± 0.10^∗^	1.66 ± 0.52
	*ZmIPT8*	0.84 ± 0.21	1.91 ± 0.50	0.68 ± 0.17	0.63 ± 0.15^∗^	0.41 ± 0.13^∗^
	*ZmIPT9*	1.05 ± 0.29	n.d.	0.58 ± 0.37	n.d.	0.47 ± 0.24^∗^
	*ZmIPT7*	0.74 ± 0.34	0.55 ± 0.31^∗^	0.68 ± 0.35	1.38 ± 0.41	6.24 ± 1.47^∗^
	*ZmIPT4*	n.d.	3.28 ± 0.05^∗^	0.55 ± 0.28	1.26 ± 0.45	1.21 ± 0.34
	*ZmCKX1*	1.08 ± 0.13	14.1 ± 5.94^∗^	118 ± 58.2^∗^	6.11 ± 0.81^∗^	145 ± 85.9^∗^
***F. verticillioides***	*ZmIPT5*	1.01 ± 0.34	1.78 ± 0.57	1.39 ± 0.31	0.31 ± 0.04^∗^	2.33 ± 0.49^∗^
	*ZmIPT3b*	0.72 ± 0.17	1.22 ± 0.33	1.25 ± 0.06	0.51 ± 0.10^∗^	0.65 ± 0.10^∗^
	*ZmIPT6*	1.01 ± 0.54	4.95 ± 2.36^∗^	2.51 ± 0.51^∗^	0.69 ± 0.23^∗^	11.2 ± 6.87^∗^
	*ZmIPT8*	1.24 ± 0.37	1.40 ± 0.40	0.95 ± 0.22	0.47 ± 0.06^∗^	0.70 ± 0.07^∗^
	*ZmIPT9*	0.92 ± 0.45	n.d.	0.43 ± 0.21^∗^	n.d.	0.24 ± 0.16^∗^
	*ZmIPT7*	1.10 ± 0.40	0.30 ± 0.22^∗^	0.32 ± 0.18^∗^	0.94 ± 0.10	6.51 ± 1.71^∗^
	*ZmIPT4*	n.d.	0.95 ± 0.28	1.27 ± 0.58	1.56 ± 0.56	0.96 ± 0.32
	*ZmCKX1*	0.60 ± 0.02^∗^	2.10 ± 0.64^∗^	28.7 ± 12.5^∗^	0.68 ± 0.12^∗^	2.01 ± 0.90
***F. fujikuroi***	*ZmIPT5*	1.26 ± 0.21	2.16 ± 0.72	0.58 ± 0.34	2.03 ± 0.84	0.89 ± 0.41
	*ZmIPT3b*	0.87 ± 0.18	3.50 ± 1.49^∗^	0.70 ± 0.29	1.77 ± 0.70	0.68 ± 0.36
	*ZmIPT6*	1.43 ± 0.44	5.01 ± 2.32^∗^	0.61 ± 0.35	1.53 ± 0.69	1.68 ± 0.32
	*ZmIPT8*	0.89 ± 0.17	1.24 ± 0.31	0.94 ± 0.25	0.39 ± 0.29^∗^	2.36 ± 1.09
	*ZmIPT9*	0.68 ± 0.39	n.d.	0.48 ± 0.24^∗^	n.d.	n.d.
	*ZmIPT7*	1.13 ± 0.32	1.67 ± 0.38	0.28 ± 0.20^∗^	1.93 ± 0.85	5.31 ± 2.41^∗^
	*ZmIPT4*	n.d.	0.20 ± 0.13^∗^	1.08 ± 0.47	n.d.	0.45 ± 0.25^∗^
	*ZmCKX1*	0.74 ± 0.10^∗^	2.59 ± 0.41^∗^	1.10 ± 0.27	3.59 ± 1.87	2.06 ± 0.62

**In the shoots of maize seedlings**						

	**Genes**	**1 dpi**	**3 dpi**	**4 dpi**	**6 dpi**	**10 dpi**

***F. mangiferae***	*ZmIPT5*	–	–	0.79 ± 0.10	2.41 ± 1.05	9.98 ± 2.71^∗^
	*ZmIPT8*	–	–	1.06 ± 0.32	9.81 ± 4.59^∗^	0.65 ± 0.41
	*ZmIPT3b*	–	–	1.02 ± 0.57	4.53 ± 2.14^∗^	0.64 ± 0.35
	*ZmIPT6*	–	–	3.20 ± 1.08^∗^	74.1 ± 37.8^∗^	2.63 ± 1.21
	*ZmIPT7*	–	–	0.22 ± 0.31	14.1 ± 6.91^∗^	0.47 ± 0.27^∗^
	*ZmCKX1*	–	–	7.84 ± 2.68^∗^	85.6 ± 12.9^∗^	36.7 ± 10.2^∗^
***F. verticillioides***	*ZmIPT5*	–	–	0.88 ± 0.24	1.68 ± 0.63	26.8 ± 5.78^∗^
	*ZmIPT8*	–	–	0.57 ± 0.14^∗^	1.43 ± 0.85	1.46 ± 0.41
	*ZmIPT3b*	–	–	2.37 ± 1.04	1.06 ± 0.50	1.70 ± 0.37
	*ZmIPT6*	–	–	0.91 ± 0.45	12.9 ± 6.32^∗^	18.6 ± 4.8^∗^
	*ZmIPT7*	–	–	4.60 ± 2.22^∗^	1.22 ± 0.57	0.38 ± 0.52
	*ZmCKX1*	–	–	24.1 ± 8.21^∗^	17.4 ± 10.7^∗^	13.3 ± 2.89^∗^
***F. fujikuroi***	*ZmIPT5*	–	–	1.70 ± 0.75	1.39 ± 0.65	12.5 ± 3.75^∗^
	*ZmIPT8*	–	–	0.98 ± 0.31	0.38 ± 0.30^∗^	0.74 ± 0.38
	*ZmIPT3b*	–	–	0.97 ± 0.35	0.97 ± 0.47	1.57 ± 0.68
	*ZmIPT6*	–	–	2.43 ± 1.18	0.80 ± 0.62	5.70 ± 3.43
	*ZmIPT7*	–	–	0.62 ± 0.42	0.67 ± 0.62	0.58 ± 0.29
	*ZmCKX1*	–	–	1.28 ± 0.30	3.09 ± 1.08^∗^	7.05 ± 3.58^∗^


*Relative expression was compared to mock-treated seedlings. Mean values with standard deviations of six biological replicates are given. ^∗^ indicates significant difference between mock and *Fusarium*-infected tissue according to Student’s unpaired *t*-tests at *p* ≤ 0.05 (*n* = 6).*

Analysis of expression of seven maize *IPTs* (*ZmIPT3b, ZmIPT4, ZmIPT5, ZmIPT6, ZmIPT7, ZmIPT8* and *ZmIPT9*) potentially involved in CK metabolism in seedling roots or shoots was investigated (Table 6). In roots, transcripts for all seven *IPT*s were detected. At almost every time point after 1 dpi, transcripts for multiple IPTs accumulated significantly more in infected roots compared to uninfected roots (Table 6). By far, *ZmIPT7* transcripts accumulated the most at 10 dpi in roots infected by all three fungi. In the shoots, transcripts for five of the seven *ZmIPT* genes were detected (Table 6). The most significant accumulation was observed in seedlings infected by *F. mangiferae* at 6 dpi where transcripts of four *IPTs* (*ZmIPT3b, ZmIPT6, ZmIPT7* and *ZmIPT8*) were present 4.5- to 74-fold greater than mock-treated seedlings or leaves of seedlings infected by the other two *Fusarium* species. At 10 dpi, *ZmIPT* levels had decreased to levels detectable in mock-treated leaves with the exception of *ZmIPT5*. The expression of two out of five CK biosynthesis genes (*ZmIPT5* and *ZmIPT6*) were significantly elevated in leaves of seedlings infected for 10 dpi by *F. verticillioides* and to a lower extent *F. fujikuroi*.

The increase of expression maize *IPT* genes appeared to be associated with the concomitant increase in *ZmCKX1* gene expression in the roots, as well as in the shoots of seedlings infected by *Fusarium* with a peak at 3–4 dpi and 6 dpi, respectively (Table 6). Seedlings infected by *F. mangiferae* showed the highest *ZmCKX1* expression followed by *F. fujikuroi* and then *F. verticillioides*.

### Effect of Fungal Auxin/Cytokinin Modification on the Pathogenicity of *Fusarium* in Maize Seedlings

In order to better understand the role of fungal auxin IAA and CKs in pathogenicity, we examined strains of *Fusarium* with altered IAA and CK metabolism. *F. verticillioides, F. proliferatum* ET1, *F. mangiferae* and *F. fujikuroi* strains over-expressing genes encoding enzymes for IAA synthesis (*IAAH* and *IAAM*) and for CK synthesis (*IPTLOG1* and *IPTLOG2*, and P450-1 and P450-2) were generated previously (Niehaus et al., 2016). For this study, we created *F. mangiferae* single and double deletion mutants of *IPTLOG1* and *tRNA-IPT*, encoding a putative tRNA-IPT critical for a tRNA-decay-based CK biosynthetic pathway (FMAN_10018). We also created *F. mangiferae IPTLOG1* and *IPTLOG2* overexpression mutants as well as a *F. mangiferae* mutant overexpressing *IPTLOG1* from *F. fujikuroi* (Table 7).

**Table 7:** Different *F. mangiferae* mutant strains derived from MRC7560 with altered CK metabolisms and their ability to produce CKs in axenic culture.

Strain	Deletion (Δ)/overexpressed (OE) gene	Abbreviation	CK content (pmol.L^-1^ of culture filtrate)			
			
			iP-types	*t*Z-types	*c*Z-types	DHZ-types
*F. mangiferae* MRC7560	–	*Fm*	7.4 ± 6.9	17.2 ± 13.7	78.4 ± 45.0	2.45 ± 2.18
*F. mangiferae* MRC7560	Δ*IPTLOG1*	Δ*Fm_IL1*	5.5 ± 2.8	4.7 ± 3.3	47.8 ± 19.4	<LOD
*F. mangiferae* MRC7560	Δ*tRNA-IPT*	Δ*Fm_tI*	3.9 ± 2.4	10.2 ± 6.5	<LOD	<LOD
*F. mangiferae* MRC7560	Δ*IPTLOG1*/Δ*tRNA-IPT*	ΔΔ*Fm_IL1/tI*	<LOD	<LOD	<LOD	<LOD
*F. mangiferae* MRC7560	*IPTLOG1* (OE)	*Fm_FmIL1*	89.4 ± 55.3^∗^	49.0 ± 17.2	91.6 ± 38.4	398.0 ± 32.8^∗^
*F. mangiferae* MRC7560	*IPTLOG2* (OE)	*Fm_FmIL2*	11.5 ± 4.9	23.4 ± 13.8	65.7 ± 35.2	5.65 ± 2.6
*F. mangiferae* MRC7560	*FfIPTLOG1* (OE)	*Fm_FfIL1*	261.0 ± 12.0^∗^	115 ± 64.9^∗^	51.9 ± 10.9	2595.0 ± 1642.0^∗^


*Mean values of two independent cultures are given. ^∗^ indicates significant differences between WT and mutant strain according to Student’s unpaired *t*-tests at *p* ≤ 0.05 (*n* = 2). Concentrations are in pmol per 1 l of culture filtrate. iP-types, isopentenyladenine derivatives; *t*Z-types, *trans*-zeatin derivatives; *c*Z-types, *cis*-zeatin derivatives; DHZ-types, dihydrozeatin derivatives; <LOD, below the limit of detection.*

Initial analysis of 7-day-old cultures of the new strains indicated significant differences between the mutants and wild-type (Table 7). The *FmIPTLOG1* mutant presented a slight, but not significant, decrease in *t*Z-type CKs. The lack of *c*Z derivatives in the *FmtRNA-IPT* mutant indicate that the transferase encoded by this gene is responsible for most *c*Z production in *F. mangiferae*. Trace production of iP and *t*Z in this deletion mutant excluded a unique role of *FmtRNA-IPT* in CK synthesis, suggesting *de novo* CKs production is mediated by *FmIPTLOG1, FmIPTLOG2* or both genes. No CKs were detected in the *FmtRNA-IPT/FmIPTLOG1* double mutant indicating that *FmIPTLOG1* but not *FmIPTLOG2* was critical for CK production in *F. mangiferae.* This was supported by a significantly higher level of CK content in the *IPTLOG1* as compared to the *IPTLOG2* overexpression strain. Indeed, while the *FmIPTLOG2* overexpression strain did not differ from the WT strain, up-regulation of *FmIPTLOG1* led to significant accumulation of iP and DHZ. A similar increase in CK types was observed in the *F. mangiferae* strain overexpressing the *IPTLOG1* gene from *F. fujikuroi* indicating that *IPTLOG1* gene function was conserved in both species (Supplementary Table S6).

To explore whether the modification of fungal CK or IAA content can impact virulence, we examined the ability of the different IAA and CK accumulating strains to cause maize seedling blight (Supplementary Figure S1). Seed germination rate, seedling height and seedling weight were usually lower than uninoculated plants, after 20 days growth (Supplementary Table S6), except for *F. fujikuroi* infected seedlings. In almost all cases, however, such differences were not statistically significant, due likely to variability between biological replicas. Nevertheless, the results indicate that both WT and IAA/CK-gene-overexpression strains of *F. mangiferae, F. proliferatum* and *F. verticillioides* could inhibit maize seed germination and reduce the height and weight of seedlings resulting from germinated seeds. The variability between biological replicas suggest that the strains were inconsistent in their ability to cause seedling-blight symptoms. The *F. verticillioides* overexpression strains were the exception in that they caused statistically significant reductions in seedling weight compared to the control treatment (Supplementary Table S6). The most reduced height and weight (albeit not statistically significant) was observed on seedlings infected by WT strain of *F. mangiferae*, similarly to previous work (Niehaus et al., 2016). In contrast, a reduction in growth was not observed for seedlings infected with the *F. mangiferae* overexpression strains. Another difference noted was that the *F. fujikuroi* strain overexpressing the CK1 cluster gene *FfIPTLOG1* significantly impacted seedling weight (but not height) as compared to all other *F. fujikuroi* strains and the control treatment.

Because we found that infection by *F. mangiferae* induced significant changes in CK content in both roots and shoots of maize seedlings, we examined the ability of CK-over accumulating and CK-deficient strains of *F. mangiferae* to affect maize seedling growth (Table 8). Mean height of seedlings infected by all strains including WT was significantly lower and the length of the primary root was significantly shorter 10 dpi in contrast to non-infected seedlings (Table 8). The most significant change was observed in seedlings infected with the strain overexpressing *FmIPTLOG1*. Seedlings dry weight or primary root length was significantly correlated with their reduced growth (Table 8). Besides CK content, the ability of the strains to activate plant CKX activity was monitored. All strains were able to induce a significant increase of CKX activity in infected root tissue compared to uninoculated plants. Three strains, the *IPTLOG1*/*tRNA-IPT* (ΔΔFm_IL1/tI) double mutant and the two strains overexpressing the *F. mangiferae IPTLOG1* and *IPTLOG2* accumulated significantly more CKX activity than plants infected with WT (Table 8). Deletion of *IPTLOG1* or *tRNA-IPT* did not statistically significantly change CKX levels.

**Table 8:** Pathogenicity test of *F. mangiferae* (MRC7560) wild type and mutants on maize seedlings grown in a hydroponic system and their ability to induce CKX activity.

		Shoots		Root		
			
Strain	Germination (%)	Height (cm)	Weight (g)	Length (cm)	Weight (g)	CKX activity (pkat mg^-1^)
Not infected	85 ± 22^a^	21.8 ± 2.5^a^	0.78 ± 0.06^a^	7.4 ± 0.9^a^	0.23 ± 0.01^a^	0.82 ± 0.43^a^
WT	69 ± 19^ab^	18.3 ± 2.1^b^	0.58 ± 0.04^b^	4.8 ± 0.8^b^	0.15 ± 0.05^b^	47.4 ± 16.2^b^
Δ*Fm_IL1*	65 ± 14^ab^	19.2 ± 2.2^b^	0.62 ± 0.03^b^	5.4 ± 1.2^b^	0.18 ± 0.05^ab^	68.4 ± 12.1^bc^
Δ*Fm_tI*	61 ± 14^b^	19.2 ± 1.4^b^	0.63 ± 0.02^b^	5.3 ± 0.5^b^	0.17 ± 0.03^b^	53.6 ± 14.1^b^
ΔΔ*Fm_IL1/tI*	65 ± 26^ab^	18.3 ± 1.7^b^	0.50 ± 0.07^c^	4.2 ± 0.6^b^	0.14 ± 0.03^bc^	82.7 ± 15.3^c^
*Fm_FmIL1*	63 ± 18^ab^	14.7 ± 1.8^c^	0.47 ± 0.08^c^	1.9 ± 0.9^c^	0.10 ± 0.04^c^	159.2 ± 9.5^d^
*Fm_FmIL2*	68 ± 18^ab^	16.9 ± 1.5^b^	0.56 ± 0.04^b^	3.6 ± 1.0^b^	0.14 ± 0.01^b^	80.1 ± 7.1^c^


*Effect of deletion and overexpression of cytokinin biosynthetic genes (*FmIPTLOG1, FmIPTLOG2, FmtRNA-IPT*) on the ability of *Fusarium mangiferae* to alter seed germination, growth of the seedlings and induce CKX activity evaluated 10 days post inoculation. Values for all measurement are means with standard deviation from 40 seeds/plants grown in four independent containers. The CKX activity is expressed as pkat per mg of extracted proteins. Values within a column that are followed by the same letter are not statistically significantly different (ANOVA; *P* ≤ 0.1). See Table 7 for strain gene designation.*

## Discussion

Hormone signaling networks are believed to play a significant role in regulating plant–microbe interactions. The stress hormones SA, jasmonic acid and ethylene are well known to induce plant defense responses against various pathogens including *Fusarium oxysporum* (Dempsey and Klessig, 2012; Di et al., 2016). Far less is known about the involvement of the morphogenetic hormones CKs and auxins in fungal–plant interactions. In a previous comparative “omics” analysis of species of the *Fusarium fujikuroi* species complex (FFC), we found evidence that FFC species can synthesize CKs and auxins in addition to the previously described production of the morphogenetic hormone GAs (Niehaus et al., 2016). Here, we examined the accumulation of these hormones *in planta* during a *Fusarium*–host plant interaction, their metabolism and their possible contribution to disease symptoms.

### Plants Tend to Restore Hormone Homeostasis Disturbed by the Pathogen

Although fungal infections in general can alter endogenous plant hormone levels, we found that pathogen produced hormones rarely affect plant growth and development. Recently, we confirmed that only two out of five studied FFC species were able to produce GAs, despite the fact that four of the *Fusarium* genomes contain the biosynthetic ability (Niehaus et al., 2016). *F. fujikuroi’s* unique ability to produce significant quantities of GAs leads to the elongation of rice seedling stems, the classic symptom of “bakanae” disease, and supports the efficient invasion of rice tissue and penetration of plant cells by hyphae (Wiemann et al., 2013). In this study, the expression of maize and fungal genes involved in CK metabolism during infection with different *Fusarium* helped reveal why fungal hormone synthesis minimally affected maize growth. Along with expression of CK biosynthetic genes from both organisms, we detected expression of a maize gene encoding a CKX, an enzyme involved in the irreversible degradation of CKs. Thus, despite efforts by the fungus to alter CK balance in maize tissue (either directly by producing CKs or indirectly by increasing plant CK synthesis) overall levels were actively mitigated by the plant. This effort to restore hormone homeostasis was most clearly seen in *F. mangiferae* infected maize tissue where the highest levels of CKs paralleled the highest level of plant CKX expression observed.

### Cytokinins Present in *F. mangiferae* Infected Tissue Are of Plant Origin

The quantification of hormones in infected tissue revealed a significant accumulation of CKs upon *F. mangiferae* infection. The accumulation of CKs correlates with the observed retarded proliferation and elongation of maize seedlings roots infected with *F. mangiferae*, compared to roots of mock- or other *Fusarium-*treated plants. In contrast, roots infected with the four other *Fusarium* contained a slight increase in CKs suggesting that CKs contributed minimally to the disease symptoms noted.

A recent study in *Claviceps purpurea* revealed a unique biosynthetic pathway for CK production based on the IPTLOG enzyme (Hinsch et al., 2015). Here, we describe two *IPTLOG* gene homologs present in all of the *Fusarium* genomes examined. We assumed initially that the activity of one or both might contribute to the massive CK increase observed in maize tissue infected by *F. mangiferae*. To our surprise, this appeared unlikely for multiple reasons. First both *IPTLOG1* and *IPTLOG2* were either not expressed or minimally expressed during infection. Any potential impact of *IPTLOG* in CK levels seems even less likely for the other *Fusarium* as we did not detect a significant CK increase, despite detecting an increase in expression of *F. verticillioides IPTLOG2* in infected tissue. Second, *F. mangiferae IPTLOG2* is likely not functional as its predicted open reading frame contains two stop codons 71 codons downstream from the start codon. We expressed the predicted 71 amino acid protein in *E. coli* and found that it exhibited no IPT and only weak LOG activity (data not shown). We also overexpressed *IPTLOG2* in the wild-type and found that it did not lead to more CKs than the WT *in planta*. And finally, an *IPTLOG1* deletion mutant did not alter the amount of CKs that accumulated in inoculated seedlings while the overexpression mutant did lead to an increase in CK production, similarly to the *FfIPTLOG1* overexpressing strain (Niehaus et al., 2016).

The second pathway for CK production is based on tRNA decay. This pathway can contribute to the total amount of CKs produced by fungi and effect virulence as recently shown in several fungal species (Chanclud et al., 2016; Hinsch et al., 2016; Morrison et al., 2017). Here, we identified an ortholog of *tRNA-IPT* responsible for CK synthesis in *F. mangiferae*. No significant differences were observed among *F. mangiferae tRNA-IPT* mutants nor *FmtRNA-IPT/IPTLOG1* double deletion mutants in their ability to cause seedling disease symptoms or to lower plant CKX activity. Taken together, the increase in plant endogenous CK content is most likely not due to fungal synthesis but rather to activation by the fungus of maize CK biosynthetic genes by a yet unknown mechanism. This response has already been observed for two biotrophic fungi, *Magnaporthe oryzae* (Jiang et al., 2013) and *Colletotrichum graminicola* (Behr et al., 2012), which lack *IPTLOG* genes and induce a massive accumulation of CKs *in planta*. In our study, up-regulation of maize *IPT* genes both in roots and shoots during infection by any of the fusaria strongly supports this hypothesis. The accumulation of Zm*IPT* transcripts was also accompanied by the up-regulation of the *ZmCKX1* gene, involved in CK degradation.

The accumulation of CKs in plant tissue may benefit fungi through increased sink activity and attraction of assimilates, providing essential energy for fungal growth. Accordingly, fungi which primarily infect non-assimilating sink organs, flowers in the case of *F. mangiferae* and spikes in the case of *C. purpurea*, were found to induce strong accumulation of CKs. The practical consequence of the accumulation of CKs to the plant is host tissue malformation as observed in *F. mangiferae* infections which is likely due to a CK-induced increase in cell division.

### Accumulation of Active Cytokinins Might Be the Cause of Mango Flower Malformation

It has been previously suggested that the presence of CKs in infected mango tissues may be related to pathogenicity (Nicholson and van Staden, 1988; Van Staden and Nicholson, 1989). Because we revealed in the present study that infection especially by *F. mangiferae* induced accumulation of endogenous CK in maize seedling tissue, we wondered whether a same response could be observed in mango, the natural host of *F. mangiferae*. Malformed tissue of mango buds and panicles contained up to fourfold higher iP and *t*Z free bases, the two CK forms shown to efficiently activate known CK receptors (Lomin et al., 2015). Since the association of *F. mangiferae* with mango tissue is long-term, in contrast to the short-term association we studied in maize, the CK homeostasis in mango tissues seems to be balanced as the concentration of some other CK forms were lowered to make the total CK pool in infected and healthy organs equal. The elevated levels of CK free bases we observed could be crucial to maintain levels of CK signal transduction required or responsible for development of the malformed mango tissue. Recently, a whole transcriptome analysis conducted on mango tissue during a long-term interaction with *F. mangiferae* revealed a significant deregulation of 12 plant genes likely associated with *zeatin* biosynthesis. The genes were described as coding for IPTs, tRNA-IPT, CKXs and CK-specific hydroxylases (Liu et al., 2016). Similarly, crown galls induced by *Agrobacterium tumefaciens* or induced by *Rhodococcus fascians* infections require elevated levels of active CKs for the development of the malformed plant tissues (Frébort et al., 2011).

Taken together, the elevated CK in plant tissue infected by *F. mangiferae* is likely due to the induction of plant CK synthesis by the fungus by an unknown mechanism. One possibility is through the action of other phytohormones or volatiles. Intriguingly, the largest up-regulation in maize *IPT* genes was observed in the shoots 6 dpi when fungal hyphae were sparse. The plant pathogen *Alternaria alternata* has already been shown to produce volatile compounds which dramatically increase CK content in the rosette leaves of *Arabidopsis* (Sánchez-López et al., 2016). Although *F. mangiferae* can produce the volatile phytohormone ethylene, for which a role in mango tissue malformation has been hypothesized (Ansari et al., 2013, 2015), based on our transcriptomic data, ethylene-response gene expression in maize tissue infected by *F. mangiferae* was attenuated suggesting that ethylene is likely not responsible for CK elevation upon *Fusarium* infection.

### Possible Role of IAA-Aspartate in Responses to *Fusarium* Infection

Besides over-accumulating CKs, roots infected with *F. mangiferae* also contain a high level of auxin (IAA), similar to roots infected with *F. proliferatum* ET1. In *F. proliferatum* ET1, IAA accumulation was related to the activation of the IAM pathway. As *F. mangiferae* does not have a functional IAM pathway, the higher amount of free IAA might be attributed to the IPyA pathway possible via a *YUCCA* gene homolog located between the GA and CK clusters. Except for *F. proliferatum* ET1, *F. mangiferae* is the only species that up-regulated this gene upon infection (Niehaus et al., 2016). Recently, it has been shown that elevated CK levels promote auxin biosynthesis in young roots (Jones et al., 2010); while exogenously applied auxin activates CK degradation (Werner et al., 2006). These trends in mutual regulation of metabolism can be overruled by local, developmentally context-specific cues (Bielach et al., 2012). We observed a twofold increase in total auxin turnover in tissue infected by *Fusarium* strains overexpressing either of the CK clusters. Auxin–CK interactions determine several processes *in planta* and have an essential impact on root and shoot morphology (Werner et al., 2001).

Infection by fungi greatly affect the auxin status in the plant host, marked by differential regulation of genes involved in IAA synthesis and conjugation (Niehaus et al., 2016). In the present study, the ratio between free and conjugated IAA revealed large differences between root tissues infected by different species and indicated that the process of IAA conjugation or conjugate hydrolysis may be part of the maize response to *Fusarium*. Surprisingly, higher accumulation of the aspartate-conjugate (IAA-Asp) was detected in *F. mangiferae* infected roots in contrast to roots infected by other *Fusarium* or mock-treated roots. IAA-Asp is an irreversible, non-active conjugate and a precursor of IAA degradation in plants (Ludwig-Müller, 2011). Prior to degradation, IAA-Asp can promote disease development induced by several pathogens in different plant species (González-Lamothe et al., 2012). For instance, *Arabidopsis* inoculated with the fungal necrotroph *Botrytis cinerea* accumulates huge amount of IAA-Asp that seemed to promote plant susceptibility to pathogens via the transcription of virulence genes (González-Lamothe et al., 2012).

Quantification of IAA-conjugates in axenic cultures revealed that *F. mangiferae* is the only *Fusarium* tested that is not able to form IAA-Asp (Niehaus et al., 2016). This lack of endogenous IAA-Asp suggests a potentially unique role of this molecule in plant–pathogen interaction. The ability of *F. mangiferae* to induce IAA-Asp production or its retention *in planta* earlier or more efficiently than the other *Fusarium* may be related to how *F. mangiferae* interacts with its host mango. Tissue infected by *F. verticillioides*, an endophyte and pathogen of maize, also accumulated more IAA-Asp than tissue infected by the other three fusaria but to a lower extent than *F. mangiferae*. Alongside IAA-Asp accumulation, *F. verticillioides* was also the second *Fusarium* isolate beside *F. mangiferae*, which was able to extensively accumulate CKs upon infection, especially in the upper part of the plant. Hence, the observed overproduction of plant CKs can be a consequence of the accumulation of IAA-Asp in infected tissue. How *Fusarium* spp. or the plant itself perceives and responds to IAA-Asp changes, whether IAA-Asp affects other fungal virulence factors and how the plant responds to IAA-Asp changes, will require further studies.

## Conclusion

Hormones, including GAs, auxins and CKs, play an important role in some *Fusarium–*host interactions. *Fusarium* species, particularly of the FFC, evolved multiple biosynthetic pathways for their *de novo* synthesis. Over time, due likely to changing evolutionary pressures, these biosynthetic genes have been lost or gained on multiple independent occasions. The characterization of CKs in Sordariomycetes provides an example how CK production may have been selected for during fungal evolution and their interaction with a plant host. CK production by the bi-functional enzyme IPTLOG appears to be an evolutionary relic, persisting only in some species such as *Claviceps purpurea* (Hinsch et al., 2015), which was replaced by a biosynthetic pathway based on the degradation of the isoprenylated tRNA (Chanclud et al., 2016). Here, we present evidence that although *Fusarium* can synthesize CKs, increases in CK levels *in planta* during an infection is likely through fungal induced changes in plant CK biosynthesis, by a yet unknown mechanism. We also revealed the existence of an alternative biosynthetic pathway for auxin production, in addition to the IAM pathway which was originally identified in bacteria. However, despite the potential functionality of this alternative pathway, the IAM pathway is the only one substantially induced during infection of the host plant. Overall, *Fusarium* species appear much more likely to manipulate plant auxin homeostasis by hydrolysis of IAA-amino acid and sugar conjugates or perhaps, regulating enzymes participating in synthesis, than *de novo* synthesis.

## Author Contributions

PG, BT, RP, E-MN, and JV designed the research. JV, ON, AP, DT, KH, MH, VB, JO, AS, SF, LO-Y, YI, MM, and ME performed the experiments and data collection. Analysis of data was ensured by PG, UG, MtM, E-MN, JV, and VB. PG, BT, DB, and VB wrote and revised the manuscript. All authors approved the final version of the manuscript.

## Conflict of Interest Statement

The authors declare that the research was conducted in the absence of any commercial or financial relationships that could be construed as a potential conflict of interest.
