# Supplementary material for: Production and Role of Hormones During Interaction of Fusarium Species With Maize (Zea mays L.) Seedlings
Source: Front Plant Sci. 2019 Jan 11;9:1936. doi: 10.3389/fpls.2018.01936 (PMC6337686; doi:10.3389/fpls.2018.01936)

Supplementary Material

1 Production and role of hormones during interaction of *Fusarium* species with maize (*Zea mays* L.) seedlings

Josef Vrabka[1], Eva-Maria Niehaus[2], Martin Münsterkötter[3], Robert H. Proctor[4], Daren W. Brown[4], Ondřej Novák[1], Aleš Pěnčík[1], Danuše Tarkowská[1], Kristýna Hromadová[1], Michaela Hradilová[1], Jana Oklešťková[1], Liat Oren-Young[5], Yifat Idan[5], Amir Sharon[5], Marcel Maymon[6], Meirav Elazar[6], Stanley Freeman[6], Ulrich Güldener[7], Bettina Tudzynski[2], Petr Galuszka[1†], Veronique Bergougnoux*[1]

Correspondence: Veronique Bergougnoux**:** veronique.bergougnoux @upol.cz

† deceased 4th of June 2018

Table S1. Primers and Taqman probes used in this study. The name of the primer and sequence in 5´to 3´orientation are listed.

Primer	Sequence
**Primers for quantitative real-time PCR (*Fusarium*)**	
Fver IL1_F	ACGGAGGCGGCACAACT
Fver IL1_R	TCAGGCCCAGAAAGGTCAAC
Fver IL1_probe	ATGGGCGCAATCGCAAGCACTC
Fver IL2_F	GGGTCTATTTGGCGGATCTTC
Fver IL2_R	CCGCCGTAGACGAGTTTGTAG
Fver IL2_probe	TGCAGCCAAGAAGCTTGCATTTGCTC
Fman IL1_F	ACGGAGGCGGCACAACT
Fman IL1_R	TCAGGCCCAGAAAGCTCAAC
Fman IL1_probe	ATGGGCGCAATCGCAAGCACTC
Fman IL2_F	CGGGCTATTTGGCGGATCTTCGC
Fman IL2_R	CCGCCGTAGACGAGTTTGTAG
Fman IL2_probe	TGCAGCCAAGAAGCTTGCATTTGCTC
Ffuj IL1_F	GCATCTTTGGTGGTTCGTCAT
Ffuj IL1_R	ACAGACTCTGCCGAGTGACTTG
Ffuj IL1_probe	CGAGCCGGCCCATATAGAAGCCG
Ffuj IL2_F	GCTACAGTTTGCGAACGATCAA
Ffuj IL2_R	CCGCCAAATAGACCAACGA
Ffuj IL2_probe	TCCACCAATGGAACACTAAACTCTAGCCCC
Fman UBI_F	GATCCTCTTGTGCCCGAGATC
Fman UBI_R	CCGAGCTGTGGCCTCGTA
Fman UBI_probe	CCATGTCTACAAGACTGACCGACCCCG
Ffuj/Fver ACTIN_F	CATTGTCATGTCTGGTGGTACCA
Ffuj/Fver ACTIN_R	AGCAAGGGCAGTGATCTCCTT
Ffuj/Fver ACTIN_probe	CATGTACCCTGGTCTCTCCGACCGTATG
**Primers for quantitative real-time PCR (maize)**	
ZmCKX1_F	CGGTGTCGCTGCTCTTCTC
ZmCKX1_R	ATCCAGTACAAGACCTACCTGGCG
ZmCKX1_probe	TGGCGAGGCTGCAGGAGCAGAACAGGAGG
ZmIPT3b_F	GATTGCGTGCAACAGAAACG
ZmIPT3b_R	GAAGGAAACAGAGATGCCTAGGTATT
ZmIPT3b_probe	CAAGCAGCTGCCCATTTTTGCCG
ZmIPT4_F	GGCGACGCGGAAAGC

ZmIPT4_R	CGGACGCCCCACATCTT
ZmIPT4_probe	CGCCGCCATCGAGGACATCAA
ZmIPT5_F	CCGCGCCGTGCATT
ZmIPT5_R	CCGTGGAGGCAAACATGGA
ZmIPT5_probe	AGTACAGCAGCAGCATGGTCACCGC
ZmIPT6_F	CCACGGAGGTGTTCCTGAAG
ZmIPT6_R	CCCATGCTGCTGTACTCTTGTT
ZmIPT6_probe	CGCCGCGCCCTGCATTG
ZmIPT7_F	AGGCTGGAGCGACATCCA
ZmIPT7_R	CGCGCTGTGCCTTTGG
ZmIPT7_probe	CTTCAGCTCAAGGTCGGGAACGCC
ZmIPT8_F	AGGGAGGAGACTGTGAATTCGA
ZmIPT8_R	AAAATTTGAACTGTCTAGTAGTGGTGGAT
ZmIPT8_probe	TTCTTCTTTTTGCTTTTGGTTCGTCCGTTC
ZmIPT9_F	CCACGCCGTGCATCGA
ZmIPT9_R	AGCAAACACGGGCACTACTTC
ZmIPT9_probe	CGTCGTCGCTGCGGCCAAT
ZmEF1_F	TGATACCCACCAAGCCTATGGT
ZmEF1_R	CATGTCGCGGACAGCAAAC
ZmEF1_probe	AGACATTCTCCGCGTTTCCTCCCCT
ZmACT_F	GAGCCACACCGTCCCTATCTAC
ZmACT_R	CACGACCAGCAAGGTCCAA
ZmACT_probe	AGGGTACACGCTTCCTCATGCTATTCTTCG
**Primers for cloning**	
Hph-F	GTCGGAGACAGAAGATGATATTGAAGGAGC
Hph-R	GTTGGAGATTTCAGTAACGTTAAGTGGAT
Fm_IL1_5F	GTAACGCCAGGGTTTTCCCAGTCACGACGCTCTCTTAGCCGGTAAGATGCC
Fm_IL1_5R	ATCCACTTAACGTTACTGAAATCTCCAACTCCTCTACCATGAGCTTTGAGCCG
Fm_IL1_3F	CTCCTTCAATATCATCTTCTGTCTCCGACGATTGGGTCTAGGCTATGGTCG
Fm_IL1_3R	GCGGATAACAATTTCACACAGGAAACAGCCCAGGTCAAATGACTCAGAGAACC
Fm_IL1_OE_F	ATGGAGTCTACCAACCGATTTATGATTGGCCATCACATCACAATCGATCCAACC
Fm_IL1_OE_R	TAATCATACATCTTATCTACATACGCTAGACCCAATCTAGTTCCCCTTTGC

tRNA-5F	GTAACGCCAGGGTTTTCCCAGTCACGACGAGCCTTCCACATGATGGACTCGC
tRNA-5R	ATCCACTTAACGTTACTGAAATCTCCAACTACCGACACTTGCTCTCGTCGG
tRNA-3F	CTCCTTCAATATCATCTTCTGTCTCCGACAACAGTCAAACCTGCCATGCCG
tRNA-3R	GCGGATAACAATTTCACACAGGAAACAGCAGCCATCGCTGGATAACTTAGCG
**Primers for diagnostic PCR**	
pCSN44-hph-trpC-T	GGAATAGAGTAGATGCCGACCGG
pCSN44-trpC-P2	GTGATCCGCCTGGACGACTAAACC
Fm_IL1-diag-5F	TCGGAAGGTTTACAGAGCGTCTTCG
Fm_IL1-diag-3R	GGTTCTTCTGATTCAAGAACGGAATGCG
tRNA-diag-F	ACGGCCAGTGTTGATGGTGGTGG
tRNA-diag-R	CTGAAACTGACCAATGAGGACCC
FM_IL1-WT-F	CGTAGCCAAGTCCGTCCATGGCC
FM_IL1-WT-R	ATCACTCGCCATTCGAATTGCAGG
tRNA-WT-F	TGTGCTAGGATCAACAGGCACGG
tRNA-WT-R	CCTTTGTCATGTCGAGTCGTTGGC

Table S2. Gibberellin (GA) levels in roots and shoots of maize seedlings 10 days post inoculation with different *Fusarium* species. Results represent mean values with standard deviations obtained from four biological replicates; * indicates significantly different from mock-treated seedlings (Student's unpaired *t*-tests at $p \leq 0.05$; n = 4). Concentrations are in pmol per g dry weight. LOD: below the detection limit. *Fv*: *F. verticillioides ; Fp*_N: *F. proliferatum* NRRL 62905; *Fp*_E: *F. proliferatum* ET1; *Fm*: *F. mangiferae*; *Ff*: *F. fujikuroi*.

GA content in the roots of maize seedlings infected by different species of *Fusarium*						
	**Mock-treated**	***Fv***	***Fp*_N**	***Fp*_E**	***Fm***	***Ff***
**Active GAs**						
			13-non-hydroxylated			
GA_4	10.00 ± 0.81	4.55 ± 0.68*	6.38 ± 1.94*	14.3 ± 3.80*	19.3 ± 0.78*	3919 ± 565*
GA_7	0.96 ± 0.40	0.75 ± 0.12	0.53 ± 0.21	15.1 ± 12.9*	0.63 ± 0.12	1283 ± 109*
GA_{13}	0.75± 0.01	1.42 ± 0.21*	2.97 ± 0.71*	25.2 ± 0.48*	0.61 ± 0.06*	2904 ± 154*
			13-hydroxylated			
GA_1	3.79 ± 0.33	1.64 ± 0.19*	2.35 ± 0.32*	7.20 ± 0.85*	4.10 ± 1.25	104 ± 4.42*
GA_3	7.19 ± 0.60	8.90 ± 1.70	9.14 ± 1.28	11.2 ± 4.64	7.93 ± 0.48	3566 ± 392*
GA_5	4.03 ± 0.64	3.21 ± 0.23*	4.13 ± 0.73	2.23 ± 0.11*	3.67 ± 0.21	2.57 ± 0.28*
Total active GAs	*26.8 ± 1.1*	*20.5 ± 1.3**	*25.5 ± 3.4*	*75.3 ± 22.2**	*36.2 ± 1.9**	*11776 ± 666**
**Deactivation products (turnover)**						
			13-non-hydroxylated			
GA_{34}	0.92 ± 0.09	0.57 ± 0.16*	0.61 ± 0.13	0.42 ± 0.09*	1.20 ± 0.21*	4.26 ± 0.20*
GA_{51}	194 ± 38.9	174 ± 10.5	329 ± 13.8	347 ± 13.9*	672 ± 79.1*	327 ± 19.0*
			13-hydroxylated			
GA_8	2.89 ± 0.57	2.28 ± 0.16	2.37 ± 0.19	1.65 ± 0.37*	2.39 ± 0.42	3.09 ± 0.30
GA_{29}	8.97 ± 0.30	12.6 ± 1.14*	15.5 ± 0.48*	13.2 ± 0.64*	15.1 ± 1.88*	24.1 ± 1.05*
Total turnover	*233 ± 40*	*210 ± 10*	*373 ± 15**	*437 ± 28**	*727 ± 81**	*12134 ± 650**
**Precursors**						
			13-non-hydroxylated			
GA_9	24.4 ± 0.51	225 ± 14.1*	178 ± 22.7*	391 ± 31.6*	405 ± 55.5*	308 ± 30.3*
GA_{12}	321 ± 80.8	252 ± 22.3	426 ± 48.6*	437 ± 78.6	498 ± 14.8*	412 ± 15.7*
GA_{15}	0.94 ± 0.30	0.54 ± 0.12*	<LOD	1.49 ± 0.46	2.88 ± 0.65*	6.97 ± 0.80*
GA_{24}	3.99 ± 0.64	1.68 ± 0.13*	2.06 ± 0.30*	2.67 ± 0.69*	3.15 ± 0.99	340 ± 49.2*
			13-hydroxylated			
GA_{19}	3.67 ± 0.30	3.73 ± 0.97	4.11 ± 0.36	3.59 ± 0.98	3.90 ± 0.70	12.2 ± 1.11*
GA_{20}	2.46 ± 0.09	2.45 ± 0.09	2.49 ± 0.33	1.35 ± 0.18*	3.66 ± 0.59*	0.77 ± 0.13*
GA_{44}	6.10 ± 1.63	5.99 ± 0.33	3.31 ± 0.83*	1.99 ± 0.18*	4.70 ± 0.82	46.1 ± 9.00*
GA_{53}	4.40 ± 0.93	1.69 ± 0.21*	3.72 ± 0.69	3.83 ± 0.54	7.06 ± 0.84*	33.0 ± 0.43*
Total precursors	*367.0 ± 81.1*	*492.5 ± 35.2**	*619.7 ± 72.4**	*843.2 ± 100.5**	*928.2 ± 59.7**	*1159.6 ± 59.2**

GA content in the shoots of maize seedlings infected by different species of *Fusarium*						
	**Mock-treated**	***Fv***	***Fp*_N**	***Fp*_E**	***Fm***	***Ff***
**Active GAs**						
			13-non-hydroxylated			
GA_4	10.4 ± 0.91	3.86 ± 0.06*	18.8 ± 5.37*	11.2 ± 5.26	17.5 ± 1.35*	74.3 ± 7.98*
GA_7	0.58 ± 0.06	0.46 ± 0.01*	1.79 ± 0.82*	2.56 ± 1.80*	0.77 ± 0.12*	53.1 ± 4.98*
GA_{13}	0.57 ± 0.04	1.10 ± 0.04*	5.23 ± 1.39*	2.68 ± 1.04*	0.46 ± 0.07*	83.7 ± 16.6*
			13-hydroxylated			
GA_1	4.30 ± 0.79	0.31 ± 0.02*	20.0 ± 1.33*	1.32 ± 0.08*	1.47 ± 0.09*	9.87 ± 0.96*
GA_3	9.95 ± 0.54	7.68 ± 1.27*	18.7 ± 3.27*	10.7 ± 3.09	7.21 ± 0.89*	102 ± 9.06*
GA_5	1.55 ± 0.11	2.30 ± 0.19*	6.77 ± 0.45*	6.69 ± 0.39*	2.68 ± 0.20*	2.44 ± 0.31*
Total active GAs	*27.4 ± 0.4*	*15.7 ± 1.5**	*71.4 ± 7.7**	*35.2 ± 9.5*	*30.1 ± 2.4*	*325 ± 33**
**Deactivation products (turnover)**						
			13-non-hydroxylated			
GA_{34}	0.77 ± 0.11	0.80 ± 0.24	1.31 ± 0.09	1.38 ± 0.91	0.78 ± 0.19	0.76 ± 0.07
GA_{51}	134 ± 20.9	150 ± 0.49	210 ± 11.9*	74.0 ± 14.6*	55.4 ± 4.47*	204 ± 43.8*
			13-hydroxylated			
GA_8	3.58 ± 0.18	2.14 ± 0.54*	3.35 ± 1.04	2.25 ± 0.40*	4.26 ± 0.90	3.34 ± 0.15
GA_{29}	14.6 ± 0.63	24.6 ± 0.29*	18.1 ± 0.71*	30.7 ± 1.63*	18.4 ± 1.03*	18.6 ± 0.72*
Total turnover	*180.0 ± 21.5*	*194.0 ± 2.0*	*304.4 ± 20.5**	*143.5 ± 26.6**	*108.9 ± 7.4**	*552.2 ± 67.6**
**Precursors**						
			13-non-hydroxylated			
GA_9	235 ± 33.3	70.0 ± 8.34*	96.5 ± 60.0*	10.7 ± 0.69*	61.6 ± 18.6*	100 ± 6.36*
GA_{12}	347 ± 30.9	230 ± 15.6*	484 ± 24.6*	404 ± 7.76	415 ± 33.2*	430 ± 92.1
GA_{15}	2.67 ± 0.18	4.16 ± 0.15*	2.60 ± 0.83	1.16 ± 0.41*	7.06 ± 0.57*	1.42 ± 0.19*
GA_{24}	3.31 ± 0.57	3.91 ± 0.91	4.11 ± 0.74	6.50 ± 0.78*	2.81 ± 0.90	14.6 ± 13.8
			13-hydroxylated			
GA_{19}	14.2 ± 1.29	10.2 ± 0.29*	8.29 ± 1.64*	13.3 ± 0.31	14.6 ± 0.28	12.1 ± 0.74*
GA_{20}	4.09 ± 0.89	5.10 ± 1.02	3.00 ± 0.28*	4.21 ± 0.59	4.60 ± 1.11	4.00 ± 1.06
GA_{44}	14.8 ± 1.48	4.65 ± 0.77*	8.96 ± 1.70*	20.6 ± 5.65	6.06 ± 1.75*	7.4 ± 0.94*
GA_{53}	15.9 ± 0.75	12.1 ± 0.22*	18.3 ± 1.21*	24.4 ± 0.87*	18.8 ± 0.88*	64.6 ± 7.58*
Total precursors	*636.1 ± 61.4*	*339.9 ± 27.0**	*625.7 ± 75.9*	*484.9 ± 56.1**	*530.8 ± 45.2*	*634.7 ± 86.7*

Table S3. Auxin levels in roots of maize seedlings infected by different *Fusarium* species 10 days post inoculation. Mean values with standard deviations obtained from three biological replicates are presented; * indicates significant differences between mock and *Fusarium*-infected tissue according to Student's unpaired *t*-tests at $p \leq 0.05$ (n = 3). Concentrations of IAA and its metabolites are in pmol per g dry weight. Abbreviations: *Fv*: *F. verticillioides;* Fp_N: *F. proliferatum* NRRL62905; *Fp*_E: *F. proliferatum* ET1; *Fm*: *F. mangiferae*; *Ff*: *F. fujikuroi*; *Ff*_IAA: *Ff* strain overexpressing *IaaM* and *IaaH* genes; *Fm*_IAA: *Fm* strain overexpressing *IAAM* and *IAAH* genes; IAA: indole-3-acetic acid; IPyA: indolepyruvic acid; IAM: indole-3-acetamide; IAA-Glc: IAA-glucose; oxIAA: 2-oxindole-3-acetic acid; oxIAA-Glc: oxIAA-glucose; IAA-Asp: IAA-aspartate; IAA-Glu: IAA-glutamate.

	**Mock-treated**	***Fv***	***Fp*_N**	***Fp*_E**	***Fm***	***Ff***	***Fp*_E_IAA**	***Ff*_IAA**
**Active**								
IAA	590 ± 106	1501 ± 958	1004 ± 421	1520 ± 618	3723 ± 848*	473 ± 88	21234 ± 19574	57402 ± 24206*
**Precursors**								
IPyA	6774 ± 1739	7430 ± 632	6180 ± 2337	9831 ± 3102	13158 ± 4700*	4785 ± 3298	1072 ± 676*	18330 ± 7808
IAM	7.4 ± 0	7.5 ± 0.6	7.6 ± 2.7	1002 ± 989	n.d.	n.d.	34520 ± 16441*	58907±34865*
Total precursors	*6782 ± 2041*	*7435 ± 707*	*6188 ± 2595*	*10820 ± 3955*	*13158 ± 4700**	*4784 ± 3508*	*35152 ± 16119**	*77237 ± 34802**
**Storage**								
IAA-Glc	4833 ± 1603	261 ± 33*	593 ± 0	574 ± 37*	239 ± 68*	888 ± 126*	n.d.	n.d.
**Degradation (turnover)**								
oxIAA	4602 ± 380	3119 ± 61*	4218 ± 1355	2912 ± 259*	4242 ± 435	2170 ± 196*	9353 ±4240	15785 ± 3855*
IAA-Asp	284 ± 59	3344 ± 2035	878 ± 108*	533 ± 77*	4128 ± 619*	506 ± 101*	868 ± 617	2595±544*
IAA-Glu	80 ± 7	365 ± 219	348 ± 68*	202 ± 87	622 ± 46*	171 ± 47*	946 ± 1260	181 ± 57*
oxIAA-Glc	878 ± 501	547 ± 40	460 ± 0	1305 ± 34	621 ± 280	884 ± 377	n.d.	n.d.
Total turnover	*11267 ± 2582*	*9136 ± 2220*	*6799 ± 1304**	*7047 ± 766**	*9734 ± 1526**	*5093 ± 876**	*32401 ± 25657*	*75963 ± 20478**
**Total auxins**	**18049 ± 3577**	**14872 ± 2485**	**12987 ± 3518**	**17867 ± 3670**	**31325 ± 3753***	**9877 ± 4122**	**-**	**-**

Table S4. Cytokinin levels in roots (A) and shoots (B) of maize seedlings grown in soil for 10 days infected by different *Fusarium* strains. Mean values with standard deviations obtained from three biological replicates (independent seedlings) are presented; * indicates significant differences between mock and *Fusarium*-infected tissue according to Student's unpaired t-tests at P ≤ 0.05 (n = 3). Concentrations of cytokinins are in pmol per g dry weight. Cytokinins were quantified by the UPLC/MS method. iP: isopentenyladenine; iPR, isopentenyladenosine; iP9G: iP *N9*-glucoside; *t*Z: *trans*-zeatin; *t*ZR: *t*Z riboside; *t*Z9G: *t*Z *N9*-glucoside; *t*ZOG: *t*Z *O*-glucoside; *t*ZROG: *t*ZR *O*-glucoside; *c*Z: *cis*-zeatin; *c*ZR: *c*Z riboside; *c*Z9G: *c*Z *N9*-glucoside; *c*ZOG: *c*Z *O*-glucoside; *c*ZROG, *c*ZR *O*-glucoside; LOD: below the limit of detection; Fp_N: *F. proliferatum* 62905; *Fp*_E: *F. proliferatum* ET1; *Fm*: *F. mangiferae*; *Ff*: - *F. fujikuroi*; *Fv: F. verticillioides; Ff*_IL2: *Ff* strain overexpressing *IPTLOG2* gene; *Ff*_IL2P2: *Ff* strain overexpressing *IPTLOG2* and *P450-2* genes.

A	Mock-treated	*Fv*	*Fp*_N	*Fp*_E	*Fm*	*Ff*	*Ff*_IL2	*Ff*_IL2P2
iP	21.6 ± 3.8	25.4 ± 7.4	23.6 ± 8.1	14.7 ± 4.0	75.5 ± 22.4*	21.1 ± 4.8	3677 ± 1284*	56.3 ± 12.4*
iPR	0.21 ±0.04	1.59 ± 0.35*	1.10 ± 0.26*	0.52 ± 0.17*	0.52 ± 0.16*	0.47 ± 0.16	3.38 ± 1.23*	0.67 ± 0.18*
iP9G	298 ± 33.1	387 ± 20.6*	304 ± 65.9	224 ± 67.5	579 ± 181*	296 ± 52.1	6 959 ± 2 282*	982 ± 317*
Total iP	*320 ± 36*	*414 ± 27**	*329 ± 73*	*240 ± 69*	*655 ± 161**	*317 ± 56*	*10639 ± 3498**	*1039 ± 318**
*t*Z	7.41 ± 1.55	3.55 ± 0.51*	5.36 ± 0.85	4.12 ± 1.08*	394 ± 101*	3.56 ± 0.38*	74.8 ± 25.2*	689 ± 229*
tZOG	12.2 ± 3.03	28.1 ± 7.01*	10.7 ± 2.09	9.21 ± 1.33	43.7 ± 12.7*	9.94 ± 2.75	10.7 ± 3.66	226 ± 53.5*
*t*ZR	1.67 ± 0.11	2.11 ± 0.57	1.06 ± 0.27*	1.10 ± 0.21*	2.80 ± 0.55*	1.11 ± 0.34*	9.10 ± 1.64*	15.1 ± 3.18*
*t*ZROG	1.26 ± 0.12	3.94 ± 0.72*	1.18 ± 0.37	1.58 ± 0.25	1.96 ± 0.51	1.05 ± 0.35	0.97 ± 0.24	1.99 ± 0.29*
*t*Z9G	193 ± 26.8	265 ± 52.9	171 ± 43.9	155 ± 50.4	576 ± 204*	194 ± 59.8	269 ± 93.3	3 345 ± 473*
Total tZ	*215 ± 28*	*303 ± 56*	*189 ± 46*	*171 ± 53*	*1018 ± 276**	*210 ± 63*	*365 ± 92**	*4277 ± 197**
*c*Z	114 ± 7.2	47.4 ± 9.4*	73.0 ± 21.4*	99.2 ± 25.0	108 ± 18.3	53.1 ± 9.2*	125 ± 20.5	102 ± 8.0
*c*ZOG	4525 ± 743	5330 ± 1217	5161 ± 1244	4607 ± 665	6993 ± 1502	4763 ± 241	7390 ± 2102	6069 ± 852
*c*ZR	100 ± 22.8	101 ± 18.8	78.3 ± 22.6	105 ± 25.9	36.4 ± 5.4*	88.1 ± 21.7	198 ± 46.5*	75.7 ± 12.7
*c*ZROG	484 ± 90.6	723 ± 126*	622 ± 112	557 ± 121	555 ± 94.5	473 ± 96.6	469 ± 120	431 ± 97.5
*c*Z9G	190 ± 3.4	18.8 ± 2.1	15.0 ± 3.1	17.4 ± 5.1	28.9 ± 2.7*	16.8 ± 0.7	397 ± 101*	35.8 ± 9.4*
*c*ZRMP	34.5 ± 0.6	32.3 ± 3.5	25.7 ± 5.7	38.0 ± 7.3	14.6 ± 0.1*	31.5 ± 7.4	34.6 ± 5.3	25.1 ± 2.9*
Total cZ	*5276 ± 784*	*6242 ± 1189*	*5975 ± 1215*	*5424 ± 766*	*7730 ± 1556*	*5425 ± 332*	*8601 ± 2264*	*6738 ± 934*
DHZ	2.84 ± 1.04	0.32 ± 0.08*	0.35 ± 0.06*	1.38 ± 0.50	151 ± 26.2*	0.42 ± 0.11*	7.51 ± 2.20*	153 ± 54.0*
DHZOG	25.6 ± 4.44	47.1 ± 2.60*	26.1 ± 8.66	31.2 ± 8.65	266 ± 76.7*	24.2 ± 7.89	49.8 ± 13.2*	743 ± 198*
DHZR	3.92 ± 1.22	3.93 ± 0.75	3.37 ± 0.95	4.46 ± 1.49	10.4 ± 2.02*	4.78 ± 1.11	19.5 ± 6.59*	17.3 ± 5.94*
DHZROG	0.67 ± 0.19	0.80 ± 0.26	0.26 ± 0.07*	1.44 ± 0.31	4.28 ± 0.78*	0.24 ± 0.02	0.49 ± 0.14	3.09 ± 0.63*
DHZ9G	3.05 ± 0.23	5.83 ± 0.97*	5.28 ± 1.73	3.53 ± 1.07	139 ± 42.8*	3.32 ± 0.77	53.5 ± 11.3*	611 ± 130*
Total DHZ	*35.8 ± 2.5*	*58.0 ± 4.4**	*35.4 ± 9.5*	*42.0 ± 11.3*	*572 ± 89**	*32.9 ± 9.1*	*131 ± 29**	*1 527 ± 341**

B	Mock treated	*Fv*	*Fp*_N	*Fp*_E	*Fm*	*Ff*
iP	27 ± 7	27 ± 6	163 ± 50*	107 ± 28*	135 ± 40*	145 ± 42*
iPR	0.61 ± 0.06	5.34 ± 0.00	<LOD	<LOD	1.39 ± 0.04*	2.43 ± 0.60*
iP9G	4.70 ± 1.35	2.14 ± 0.26	3.65 ± 1.21	4.54 ± 1.18	40.5 ± 9.30*	3.76 ± 0.87
Total iP	*31.8 ± 7.7*	*30.7 ± 6.1*	*167 ± 50**	*112 ± 28**	*177 ± 31**	*152 ± 42**
*t*Z	6.11 ± 1.21	16.8 ± 4.86*	4.02 ± 1.26	5.31 ± 1.65	66.6 ± 19.1*	4.70 ± 1.36
tZOG	3.01 ± 0.62	5.46 ± 1.24	1.57 ± 0.47	4.55 ± 0.30	82.5 ± 14.4*	8.67 ± 0.79*
*t*ZR	0.09 ± 0.03	2.18 ± 0.77*	1.17 ± 0.23*	0.51 ± 0.06*	3.72 ± 0.73*	2.22 ± 0.61*
*t*ZROG	0.11 ± 0.03	0.49 ± 0.10*	<LOD	0.26 ± 0.09*	5.58 ± 1.31*	1.98 ± 0.61*
*t*Z9G	8.82 ± 1.02	6.27 ± 1.99	9.45 ± 1.21	11.5 ± 2.75	397 ± 119*	6.72 ± 1.41
Total tZ	*17.2 ± 1.5*	*29.4 ± 5.1**	*16.2 ± 0.2*	*20.6 ± 5.4*	*556 ± 106**	*21.4 ± 5.6*
*c*Z	75.9 ± 5.9	84.8 ± 15.9	94.5 ± 31.0	49.2 ± 6.7*	76.0 ± 18.9	749 ± 213*
*c*ZOG	4775 ± 983	3256 ± 97*	4062 ± 239	4016 ± 577	8571 ± 953*	1126 ± 261*
*c*ZR	24.7 ± 7.7	47.9 ± 13.0*	37.1 ± 9.3	19.6 ± 3.7	25.6 ± 5.1	391 ± 106*
*c*ZROG	136 ± 8.3	81.3 ± 20.1*	91.3 ± 7.2*	104 ± 20.4	397 ± 63.1*	21.1 ± 4.2*
*c*Z9G	00.6 ± 0.1	0.4 ± 0.1	0.4 ± 0.1*	1.5 ± 0.4*	20.4 ± 5.7*	1.6 ± 0.2*
*c*ZRMP	19.5 ± 3.9	18.1 ± 0.8	26.5 ± 2.8	24.6 ± 8.5	21.5 ± 7.5	58.7 ± 12.9*
Total cZ	*5030 ± 980*	*3482 ± 103**	*4303 ± 227*	*4214 ± 579*	*9110 ± 1035**	*2327 ± 293**
DHZ	0.38 ± 0.10	2.92 ± 0.82*	0.28 ± 0.11	0.54 ± 0.16	41.6 ± 10.5*	5.37 ± 1.07*
DHZOG	36.4 ± 9.97	10.6 ± 1.20*	29.1 ± 8.00	45.1 ± 5.19	2 431 ± 683*	19.5 ± 4.80*
DHZR	1.50 ± 0.15	2.06 ± 0.41	2.17 ± 0.62	1.59 ± 0.26	128 ± 46.4*	13.6 ± 2.47*
DHZROG	0.50 ± 0.16	0.70 ± 0.15	0.37 ± 0.05	0.55 ± 0.17	30.0 ± 7.12*	5.77 ± 1.98*
DHZ9G	0.49 ± 0.06	1.09 ± 0.36*	0.41 ± 0.12	1.32 ± 0.40*	91.1 ± 26.3*	1.04 ± 0.24*
Total DHZ	*39.3 ± 10.2*	*17.4 ± 1.7**	*32.3 ± 7.7*	*49.1 ± 5.8*	*2 721 ± 630**	*45.3 ± 9.0*

2 **Table S5. Cytokinin metabolite profile of heathy and malformed mango reproductive organs.** Each sample was measured in two technical replicates. Cytokinins were quantified by the UPLC/MS method, and concentrations are expressed in pmol per g dry weight. iP: isopentenyladenine; iPR: isopentenyladenosine; iP9G: iP N9-glucoside; *t*Z: *trans*-zeatin; *t*ZR: *t*Z riboside; *t*ZOG: *t*Z O-glucoside; *t*ZROG: *t*ZR O-glucoside; *t*Z9G: *t*Z N9-glucoside; *c*Z: *cis*-zeatin; *c*ZR: *c*Z riboside; *c*ZOG: *c*Z O-glucoside; *c*ZROG: *c*ZR O-glucoside; *c*Z9G: *c*Z N9-glucoside; DHZ: dihydrozeatin; DHZR: DHZ riboside; DHZ9R: DHZ N9-glucoside; < LOD: below the limit of detection.

	***t*Z-types**				
	***t*Z**	***t*ZOG**	***t*ZR**	***t*ZROG**	***t*Z9G**
Young malformed panicle	18.37 ± 3.70	5.92 ± 1.70	2.54 ± 0.21	6.20 ± 0.44	1.77 ± 0.39
Young healthy panicles	7.47 ± 0.07	2.13 ± 0.22	16.24 ± 1.96	4.37 ± 1.01	2.38 ± 0.64
Swollen diseased buds	12.63 ± 0.57	8.06 ± 0.60	38.48 ± 2.24	24.62 ± 1.37	10.59 ± 1.78
Normal buds	5.51 ± 0.79	3.84 ± 0.09	6.45 ± 0.65	7.43 ± 0.04	7.02 ± 1.53
Mature malformed panicle	9.84 ± 0.38	12.35 ± 0.84	5.90 ± 0.65	8.79 ± 0.06	1.53 ± 0.05
Mature healthy panicles	2.26 ± 0.31	19.39 ± 2.80	10.97 ± 1.44	2.00 ± 0.10	1.36 ± 0.09
Malformed vegetative	8.84 ± 1.58	3.88 ± 0.39	6.48 ± 1.13	7.36 ± 0.18	7.11 ± 1.49
	***c*Z-types**				
	***c*Z**	***c*ZOG**	***c*ZR**	***c*ZROG**	***c*Z9G**
Young malformed panicle	1.36 ± 0.16	5.01 ± 0.97	0.60 ± 0.18	0.20 ± 0.00	< LOD
Young healthy panicles	0.33 ± 0.07	<LOD	1.81 ± 0.22	0.29 ± 0.05	0.10 ± 0.02
Swollen diseased buds	0.89 ± 0.08	3.11 ± 0.10	0.90 ± 0.10	1.76 ± 0.07	1.02 ± 0.17
Normal buds	0.35 ± 0.03	0.11 ± 0.03	2.04 ± 0.10	1.59 ± 0.11	0.81 ± 0.00

Mature malformed panicle	0.77 ± 0.05	3.52 ± 0.52	1.21 ± 0.15	0.11 ± 0.00	< LOD
Mature healthy panicles	0.48 ± 0.03	<LOD	4.76 ± 0.62	0.79 ± 0.05	< LOD
Malformed vegetative	1.04 ± 0.15	4.85 ± 1.21	0.48 ± 0.04	0.58 ± 0.08	0.49 ± 0.11

DHZ-types

	DHZ	DHZOG	DHZR	DHZROG	DHZ9G
Young malformed panicle	0.74 ± 0.03	0.12 ± 0.00	1.57 ± 0.04	< LOD	0.09 ± 0.01
Young healthy panicles	0.32 ± 0.06	0.07 ± 0.00	2.14 ± 0.01	0.16 ± 0.03	0.31 ± 0.11
Swollen diseased buds	2.33 ± 0.87	0.41 ± 0.02	5.97 ± 0.33	1.19 ± 0.11	0.15 ± 0.04
Normal buds	0.36 ± 0.04	0.67 ± 0.01	7.09 ± 0.12	2.37 ± 0.04	0.13 ± 0.00
Mature malformed panicle	0.79 ± 0.08	0.17 ± 0.01	2.95 ± 0.44	< LOD	2.38 ± 0.18
Mature healthy panicles	0.36 ± 0.11	0.17 ± 0.03	4.68 ± 0.79	0.33 ± 0.03	16.75 ± 5.62
Malformed vegetative	1.06 ± 0.19	0.27 ± 0.00	1.76 ± 0.03	0.58 ± 0.09	0.11 ± 0.00

iP-types

	iP	iPR	iP9G
Young malformed panicle	141.66 ± 28.20	12.91 ± 1.79	3.71 ± 0.91
Young healthy panicles	52.09 ± 5.76	24.39 ± 1.63	1.01 ± 0.28
Swollen diseased buds	74.96 ± 18.12	25.70 ± 0.46	4.38 ± 1.16
Normal buds	23.02 ± 5.26	25.38 ± 2.63	2.45 ± 0.03
Mature malformed panicle	140.16 ± 32.41	20.74 ± 3.22	4.29 ± 0.52
Mature healthy panicles	37.81 ± 3.87	45.21 ± 1.09	1.49 ± 0.29
Malformed vegetative	86.24 ± 9.28	16.32 ± 0.11	3.50 ± 0.59

3

Table S6. Pathogenicity test of *Fusarium* strains on maize seedlings. Effect of overexpression (OE) of auxin (*IAAH* and *IAAM*) and cytokinin biosynthetic (*IPTLOG1*, *IPTLOG2*, *P4502* and *P4502*) genes from *F. proliferatum* and *F. fujikuroi*, respectively, on the ability of *Fusarium* species to alter maize seed germination and growth of the resulting seedlings.

Species	Strain	Germination[a]	Height[a] (cm)	Weight[a] (g)
F. fujikuroi	Not infected	81 ab	61.1	4.62 a
	Wild type	81 ab	83.8	4.66 a
	Ff_IAA	81 ab	77.7	5.41 a
	Ff_IL1	67 b	66.9	2.71 b
	Ff_IL2	88 a	68.5	5.95 a
	Ff _IL1P1	83 ab	73.5	5.20 a
	Ff _IL2P2	79 ab	70.9	4.95 a
F. mangiferae	Not infected	81	61.1	4.62
	Wild type	61	42.6	2.61
	Fm_IAA	73	47.9	3.38
	Fm_IL1	85	52.3	4.20
	Fm_IL2	75	55.9	3.31
	Fm_IL1P1	84	54.6	3.93
	Fm _IL2P2	77	51.2	4.10
F. proliferatum ET1	Not infected	81	61.1	4.62
	Wild type	78	49.7	3.51
	Fp_E_IAA	58	44.6	2.11
	Fp_E_IL1	70	43.7	2.69
	Fp_E_IL2	64	47.8	2.95
	Fp_E_IL1P1	69	54.9	2.99
	Fp_E_IL2P2	69	60.6	3.33
F. verticillioides	Not infected	81	61.1	4.62 a
	Wild type	77	52.1	3.27 ab
	Fv_IAA	77	49.2	2.94 b
	Fv_IL1	73	46.9	2.98 b
	Fv_IL2	71	50.6	3.01 b
	Fv_IL1P1	65	49.3	2.61 b
	Fv _IL2P2	67	43.7	2.67 b

[a] Values for all measurement are means from three independent experiments. In each experiment, 10 treated seeds were sown per pot, and there were five pots per treatment. Germination is a percentage value: the number of seedlings 20 dpi in a pot per 10 seeds sown. Height was determined by measuring the length of each seedling from the soil line to the tip of the tallest leaf. Weight was determined by cutting a seedling in a pot at the soil line. For a given species, values within a column that are followed by the same letter are not statistically significantly different (ANOVA; $P \leq 0.1$). In

columns that do not include letters after the values, none of the values are statistically significantly different.

Table S7. Different forms of cytokinins produced by *Fusarium mangiferae* mutants. Cultures of mutant strains were grown in ICI media for 7 days and cytokinins were isolated from culture filtrates by immuno-affinity columns and quantified by UPLC. Mean values of two independent cultures are given. * indicates significant differences between WT and mutant strain according to Student's unpaired *t*-tests at $p \leq 0.05$ (n = 2). Concentrations are in pmol per 1 litre of the culture filtrate. *t*Z, *trans*-zeatin derivatives; iP, isopentenyladenine derivatives; *c*Z, *cis*-zeatin derivatives; DHZ, dihydrozeatin derivatives; LOD: below the limit of detection.

strain	iP type	*t*Z type	*c*Z type	DHZ type
WT	7.42 ± 6.99	17.2 ± 13.70	78.4 ± 45.0	2.45 ± 2.18
ΔFm_IL1	5.45 ± 2.78	4.65 ± 3.25	47.8 ± 19.4	LOD
ΔFm_tI	3.85 ± 2.41	10.2 ± 6.47	LOD	LOD
ΔΔFm_IL1/tI	LOD	LOD	LOD	LOD
Fm_FmIL1	89.4 ± 55.3*	49.0 ± 17.2	91.6 ± 38.4	398 ± 32.8*
Fm_FmIL2	11.5 ± 4.92	23.4 ± 13.8	65.7 ± 35.2	5.65 ± 2.60
Fm_IL1	261 ± 12.0*	115 ± 64.9*	51.9 ± 10.9	2595 ± 1642*

Supplementary Figures

Figure S1. Phenotype of maize seedlings infected with *Fusarium* mutant strains overexpressing IAA and CK biosynthetic genes 20 days post inoculation.

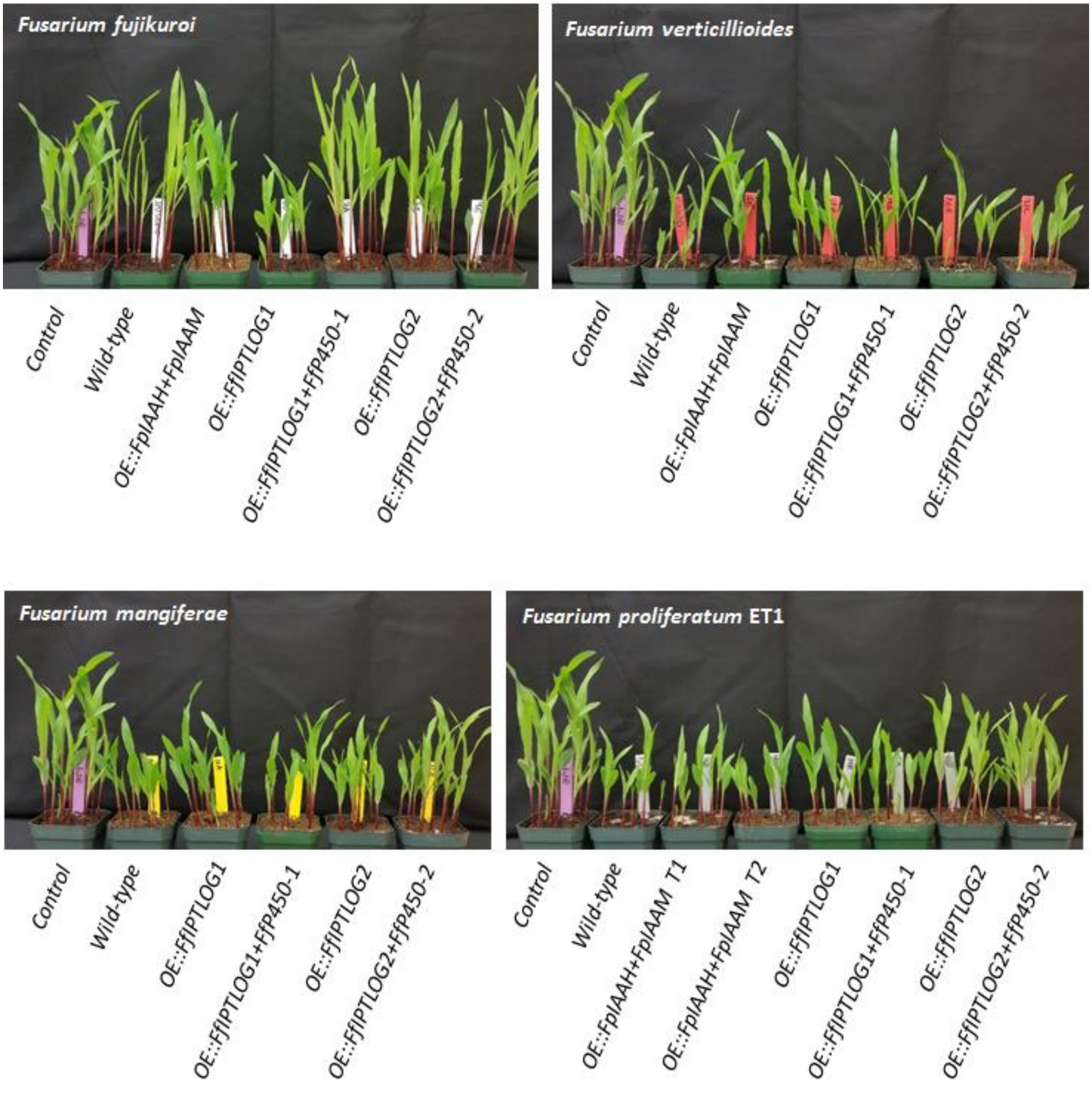

Supplement: Supplementary file 1 [file Data_Sheet_1.pdf]
